# A real-world pharmacovigilance study of FDA adverse event reporting system events for diazepam

**DOI:** 10.3389/fphar.2024.1278442

**Published:** 2024-01-24

**Authors:** Weizhen He, Yang Wang, Kaiqin Chen

**Affiliations:** ^1^ Department of Neurosurgery, Xiang’an Hospital of Xiamen University, Xia Men, Fujian, China; ^2^ Department of Ear Nose and Throat, Xiang’an Hospital of Xiamen University, Xia Men, Fujian, China

**Keywords:** diazepam, real-world data analysis, adverse event, pharmacovigilance, disproportionality analysis

## Abstract

**Background:** Diazepam, one of the benzodiazepines, is widely used clinically to treat anxiety, for termination of epilepsy, and for sedation. However, the reports of its adverse events (AEs) have been numerous, and even fatal complications have been reported. In this study, we investigated the AEs of diazepam based on real data from the U.S. Food and Drug Administration (FDA) adverse event reporting system (FAERS).

**Methods:** Disproportionality in diazepam-associated AEs was assessed through the calculation of reporting odds ratios (RORs), proportional reporting ratios (PRRs), Bayesian confidence–propagation neural networks (BCPNNs), and gamma-Poisson shrinkage (GPS).

**Results:** Among the 19,514,140 case reports in the FAERS database, 15,546 reports with diazepam as the “principal suspect (PS)" AEs were identified. Diazepam-induced AEs occurred targeting 27 system organ categories (SOCs). Based on four algorithms, a total of 391 major disproportionate preferred terms (PTs) were filtered out. Unexpectedly significant AEs such as congenital nystagmus, developmental delays, and rhabdomyolysis were noted, which were not mentioned in the drug insert.

**Conclusion:** Our study identified potential signals of new AEs that could provide strong support for clinical monitoring and risk identification of diazepam.

## Introduction

Diazepam, also known as Valium, was introduced in 1963 as a benzodiazepine anxiolytic approved by the US Food and Drug Administration (FDA) for treatment of epilepsy, anxiety, convulsions, sedation, hypnotic effects, and for central muscle relaxation. Its mechanism of action is related to facilitation of gamma-aminobutyric acid (GABA) release through binding to the central benzodiazepine receptor or facilitation of synaptic transmission function (Wang et al., 2020).

As a long-acting benzodiazepine, diazepam possesses a half-life of 24–36 h. Upon entering the body, it operates through gamma-aminobutyric acid type A (GABAA) receptors, increasing the receptors’ affinity for GABA (an inhibitory neurotransmitter) and enhancing GABA activity. This process slows down neurotransmission, resulting in sedative and anxiolytic effects (Campo-Soria et al., 2006). Due to its potent and effective pharmacological effects, diazepam has found extensive clinical use in various departments, including the intensive care unit, emergency department, neurosurgery, and neurology. However, many unexpected AEs may occur in patients using diazepam due to individual patient differences.

A therapeutic dose of diazepam administered to healthy controls has been shown to induce significant impairment of psychiatric alertness and cognitive performance, without notable effects on respiration (Mak et al., 1993). Similarly, relevant studies have demonstrated that the effects of benzodiazepines on cognitive performance are primarily observed in low doses sufficient to severely impair the driving ability (Verster et al., 2002). Moreover, benzodiazepines have also been shown to have an impact on memory, particularly on newly acquired knowledge after their consumption (Verster and Volkerts, 2004). Notably, other significant AEs such as drug dependence, drug abuse, and suicide are being reported with increasing frequency (Wang et al., 2022).

The FDA Adverse Drug Event Reporting System (FAERS), established in 2012, is one of the largest pharmacovigilance databases globally, encompassing a vast number of AEs and medication errors related to drugs and therapeutic biological products. It serves the purpose of monitoring the safety of medicines after their introduction to the market (Liu et al., 2022). There are many research projects to analyze the AEs of drugs in clinical use by mining data from the FAERS database to explore unexpected adverse drug reactions (ADRs) that are not described in the drug labels.

In this study, we used disproportionate analyses to detect diazepam-related ADR signals included in the FAERS database. The study aimed to detect new and unexpected ADRs not described in the drug label.

## Data sources and methods

### Data sources

A retrospective, observational pharmacovigilance study used data from the FAERS data published by the FDA (updated quarterly). In our study, diazepam-related AE reports submitted from the first quarter of 2004 to the first quarter of 2023 (spanning a total of 77 quarters) were extracted from the FAERS database. The data were then imported into SAS 9.4, MySQL, and Excel software for cleaning and analysis.

### Data processing

**FIGURE 1:**
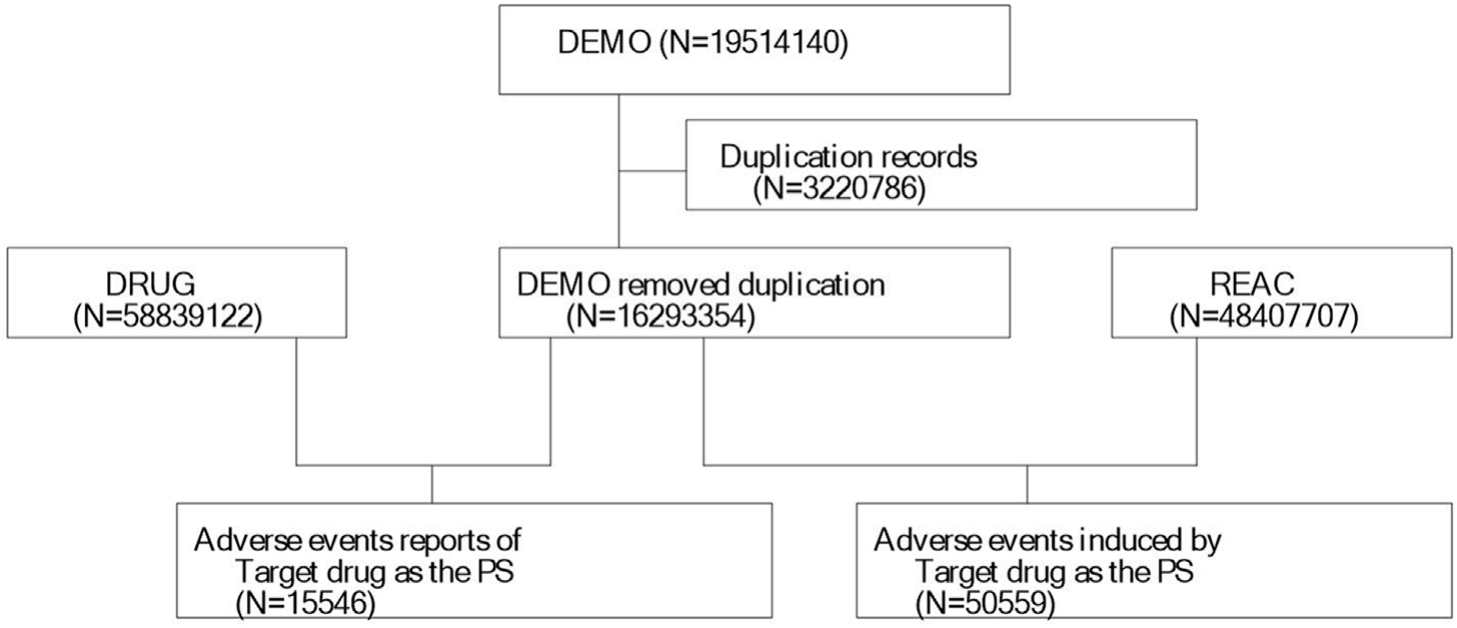
Process of selecting cases of diazepam-related AEs from the FAERS database.

We extracted 19,514,140 reports from the FAERS database, following the FDA-recommended method for removing duplicate reports. We selected the PRIMARYID, CASEID, and FDA_DT fields from the DEMO table and sorted them by CASEID, FDA_DT, and PRIMARYID. We retained the report with the largest FDA_DT value for cases with the same CASEID. Subsequently, for cases with both the same CASEID and FDA_DT, we kept the report with the largest PRIMARYID value. A list of deleted reports was included in each quarterly data package since Q1 2019, and after data deduplication, reports were excluded based on the CASEID present in the list of deleted reports. Finally, we included 16,293,354 reports for further analysis (Figure 1). MedDRA was then used to correct the preferred term (PT) names in the FAERS database and to obtain the system organ class (SOC) and preferred terms (PTs).

### Statistical analysis

Based on the rationale of disproportionality analysis and Bayesian analysis, we employed the reporting odds ratio (ROR), proportional reporting ratio (PRR), Bayesian confidence propagation neural network (BCPNN), and multi-item gamma Poisson shrinker (MGPS) algorithms to investigate the associations between the drug and the specified AEs. The equations and criteria for the four algorithms are listed in Table 1.

**TABLE 1:** Summary of algorithms.

Algorithms	Equation^a^	Criteria
ROR	$\text{ROR}=\frac{\left(a/c\right)}{\left(b/d\right)}=\frac{ad}{bc}$	a≥3, 95% CI (lower limit)>1
	$95\%\text{ CI}=e^{\ln\left(\text{ROR}\right)\pm 1.96\sqrt{\;\left(\frac{1}{a}+\frac{1}{b}+\frac{1}{c}+\frac{1}{d}\right)}}$	
PRR	$\text{PRR}=\frac{a/\left(a+b\right)}{c/\left(c+d\right)}$	a≥3, 95% CI (lower limit)>1, PRR≥2, χ2≥ 4
	95% CI = $e^{\ln\left(\text{PRR}\right)\pm 1.96\sqrt{\;\frac{1}{a}-\frac{1}{a+b}+\frac{1}{c}-\frac{1}{c+d}}}$	
	$\chi 2=\frac{\;{\left(\text{ad}-\text{bc}\right)}^{2}\left(a+b+c+d\right)}{\left(\;a+b\right)\left(a+c\right)\left(c+d\right)\left(b+d\right)}$	
BCPNN	IC = $\log_{2}\frac{p\left(x,y\right)}{p\left(x\right)p\left(y\right)}=\log_{2}\frac{a\left(a+b+c+d\right)}{\left(a+b\right)\left(a+c\right)}$	No signal (−):IC-2SD ≤ 0
	E (IC) = $\log_{2}\frac{\left(a+\gamma 11\right)\left(a+b+c+d+\alpha \right)\left(a+b+c+d+\beta \right)}{\left(a+b+c+d+\gamma \right)\left(a+b+\alpha 1\right)\left(a+c+\beta 1\right)}$	Weak signal (+):0<IC-2SD ≤ 1.5
	V(IC) = $\frac{1}{{\left(\ln2\right)}^{2}}\left\{\left[\frac{\left(a+b+c+d\right)-a+\gamma -\gamma 11}{\left(a+\gamma 11\right)\left(1+a+b+c+d+\gamma \right)}\right]+\left[\frac{\left(a+b+c+d\right)-\left(a+b\right)+\alpha -\alpha 1}{\left(a+b+\alpha 1\right)\left(1+a+b+c+d+\alpha \right)}\right]+\left[\frac{\left(a+b+c+d\right)-\left(a+c\right)+\beta -\beta 1}{\left(a+c+\beta 1\right)\left(1+a+b+c+d+\beta \right)}\right]\right\}$	Medium signal (++):1.5<IC-2SD ≤ 3
	$\gamma =\gamma 11\frac{\left(a+b+c+d+\alpha \right)\left(a+b+c+d+\beta \right)}{\left(a+b+\alpha 1\right)\left(a+c+\beta 1\right)}$	Strong signal (+++):IC-2SD > 3
	*IC-2SD = E(IC)-2* $\sqrt{V\left(IC\right)}$	
	p. s. α1=β1=1;α=β=2;γ11=1	
MGPS	$\text{EBGM}=\frac{a\left(a+b+c+d\right)}{\left(a+c\right)\left(a+b\right)}$	EBGM05 > 2
	$95\%\text{ CI}=e^{\ln\left(\text{EBGM}\right)\pm 1.96\sqrt{\;\left(\frac{1}{a}+\frac{1}{b}+\frac{1}{c}+\frac{1}{d}\right)\;}}$	

^a^ ROR, reporting odds ratio; a, number of reports containing both the suspect drug and the suspect adverse drug reaction; b, number of reports containing the suspect adverse drug reaction with other medications (except the drug of interest); c, number of reports containing the suspect drug with other adverse drug reactions (except the event of interest); d, number of reports containing other medications and other adverse drug reactions; CI, confidence interval; PRR, proportional reporting ratio; χ2, chi-square; BCPNN, Bayesian confidence propagation neural network; IC, information component; IC-2SD, the lower confidence interval of IC; MGPS, multi-item gamma Poisson shrinker; EBGM, empirical Bayesian geometric mean; EBGM05, the lower 95% one-sided CI of EBGM.

## Results

### General characteristics

**FIGURE 2:**
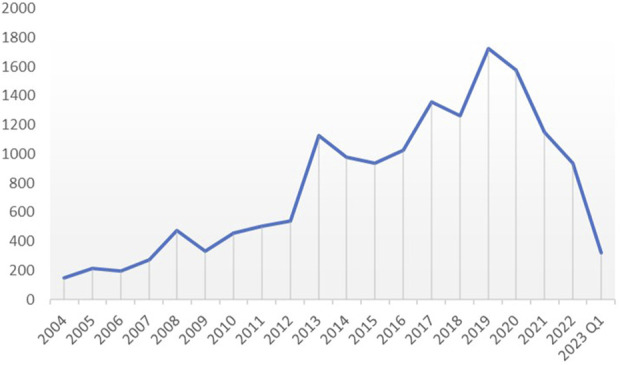
Annual trends in diazepam reporting.

We extracted all reported cases from the FAERS database from 2014 to Q1 2013, amounting to 19,514,140 cases. After deleting duplicates and screening, a total of 15,546 reports of diazepam-related adverse reactions were obtained. The reported data were analyzed in this study, and the general characteristics of the associated AEs are listed in Table 2. Among the reported AEs, the percentage of women (44.19%) was slightly higher than that of men (42.99%). Regarding age distribution, although the percentage of patients with unknown age reached 25.64%, the highest percentage of AEs of 34.24% occurred in the 18–45 year age group, followed by the 45–65 year age group with AE percentage of 24.78%. The top reporting country was the United States (40.93%), followed by France (16.13%), the United Kingdom (8.82%), Italy (5.94%), Australia (4.03%), and Canada (3.26%), among others. The most serious AE outcome was death (32.75%), whereas the other three outcomes were hospitalization (27.69%), life-threatening conditions (5.96%), and disability (1.56%). Considering the years 2004 to 2023 (Figure 2), the highest reported year was 2019 (11.08%).

**TABLE 2:** Features of reports associated with diazepam.

	Case reports (n)	Case proportion (%)
Gender		
Female	6,870	44.19
Male	6,683	42.99
Unknown	1,993	12.82
Age		
<18	795	5.11
≥18,<45	5,323	34.24
≥45,<65	3,853	24.78
≥65,<75	894	5.75
75≤	695	4.47
Unknown	3,986	25.64
Reported countries		
America	6,363	40.93
France	2,508	16.13
Britain	1,371	8.82
Italy	924	5.94
Australia	626	4.03
Canada	507	3.26
Other countries	3,247	20.89
Outcome		
Death	5,092	32.75
Hospitalization	4,304	27.69
Life-threatening	927	5.96
Disability	242	1.56
Congenital anomaly	188	1.21
Other outcomes	5,214	33.54

### Signal detection

Table 3 displays the signal intensity of diazepam at the SOC level. As depicted in the figure data, diazepam-induced AEs targeted 27 organ systems. Significant SOCs that met the criteria for at least one of the four indices were psychiatric disorders (SOC: 10,037,175, n = 9,555), injury, poisoning, and procedural complications (SOC: 10,022,117, n = 8,689); nervous system disorders (SOC: 10,029,205, n = 7,137); respiratory, thoracic, and mediastinal disorders (SOC: 10,038,738, n = 2,931); cardiac disorders (SOC: 10,007,541, n = 2,192); social circumstances (SOC: 10,041,244, n = 537); congenital, familial, and genetic disorders (SOC: 10,010,331, n = 447); and pregnancy, puerperium, and perinatal conditions (SOC: 10,036,585, n = 433). We identified that congenital, familial, and genetic disorders within the mentioned SOCs were new and valuable ADRs not listed in the diazepam drug insert.

**TABLE 3:** Signal values of reports associated with diazepam at the SOC level.

System organ class (SOC)	Case reports	ROR (95% CI)	PRR (χ2)	IC (IC025)	EBGM (EBGM05)
Psychiatric disorders^a^	9,555	3.84 (3.75, 3.93)	3.3 (16,212.08)	1.72 (1.69)	3.29 (3.22)
Injury, poisoning, and procedural complications^a^	8,689	1.91 (1.86, 1.95)	1.75 (3,098.9)	0.81 (0.77)	1.75 (1.71)
Nervous system disorders^a^	7,137	1.73 (1.69, 1.78)	1.63 (1904.46)	0.7 (0.67)	1.63 (1.59)
General disorders and administration site conditions	6,318	0.68 (0.66, 0.7)	0.72 (841.63)	−0.48 (-0.51)	0.72 (0.7)
Respiratory, thoracic, and mediastinal disorders^a^	2,931	1.24 (1.2, 1.29)	1.23 (128.95)	0.29 (0.24)	1.23 (1.18)
Investigations	2,359	0.74 (0.71, 0.77)	0.75 (211)	−0.42 (-0.48)	0.75 (0.72)
Cardiac disorders^a^	2,192	1.62 (1.55, 1.69)	1.59 (491.69)	0.67 (0.6)	1.59 (1.52)
Gastrointestinal disorders	1757	0.38 (0.37, 0.4)	0.41 (1,676.71)	−1.3 (-1.37)	0.41 (0.39)
Musculoskeletal and connective tissue disorders	1,040	0.38 (0.36, 0.41)	0.39 (1,024.67)	−1.34 (-1.43)	0.39 (0.37)
Vascular disorders	966	0.87 (0.82, 0.93)	0.88 (17.46)	−0.19 (-0.29)	0.88 (0.82)
Skin and subcutaneous tissue disorders	899	0.32 (0.3, 0.34)	0.33 (1,287.9)	−1.6 (-1.69)	0.33 (0.31)
Eye disorders	815	0.81 (0.76, 0.87)	0.82 (33.68)	−0.29 (-0.39)	0.82 (0.76)
Infections and infestations	806	0.3 (0.28, 0.32)	0.31 (1,318.52)	−1.7 (-1.8)	0.31 (0.29)
Metabolism and nutrition disorders	630	0.56 (0.52, 0.61)	0.57 (211.66)	−0.82 (-0.93)	0.57 (0.53)
Product issues	607	0.78 (0.72, 0.85)	0.78 (37.05)	−0.35 (-0.47)	0.78 (0.72)
Social circumstances^a^	537	2.31 (2.12, 2.51)	2.3 (393.44)	1.2 (1.07)	2.29 (2.11)
Immune system disorders	508	0.92 (0.85, 1.01)	0.92 (3.25)	−0.11 (-0.24)	0.92 (0.85)
Renal and urinary disorders	501	0.5 (0.46, 0.55)	0.5 (248.08)	−0.99 (-1.11)	0.51 (0.46)
Congenital, familial, and genetic disorders^a^	447	2.84 (2.59, 3.12)	2.83 (528.02)	1.5 (1.35)	2.82 (2.57)
Pregnancy, puerperium, and perinatal conditions^a^	433	1.93 (1.76, 2.13)	1.93 (193.07)	0.94 (0.8)	1.92 (1.75)
Hepatobiliary disorders	415	0.89 (0.81, 0.98)	0.89 (5.22)	−0.16 (-0.3)	0.89 (0.81)
Blood and lymphatic system disorders	279	0.33 (0.29, 0.37)	0.33 (387.26)	−1.6 (-1.77)	0.33 (0.29)
Surgical and medical procedures	259	0.39 (0.35, 0.45)	0.4 (239.53)	−1.33 (-1.51)	0.4 (0.35)
Ear and labyrinth disorders	164	0.74 (0.64, 0.87)	0.74 (14.54)	−0.43 (-0.65)	0.74 (0.64)
Reproductive system and breast disorders	130	0.27 (0.23, 0.33)	0.28 (249.82)	−1.86 (-2.1)	0.28 (0.23)
Neoplasms: benign, malignant, and unspecified (including cysts and polyps)	108	0.08 (0.06, 0.09)	0.08 (1,209.07)	−3.68 (-3.94)	0.08 (0.06)
Endocrine disorders	77	0.61 (0.49, 0.77)	0.61 (18.6)	−0.7 (-1.02)	0.62 (0.49)

^a^ indicates statistically significant signals in the algorithm; ROR, reporting odds ratio; CI, confidence interval; PRR, proportional reporting ratio; χ2, chi-square; IC, information component; IC025, the lower limit of 95% CI of the IC; EBGM, empirical Bayesian geometric mean; EBGM05, the lower limit of 95% CI of EBGM.

Supplementary Table S1 lists the 391 significantly disproportionate PTs that simultaneously complied with all four algorithms. From this list, the top 40 PTs with the highest number of reports were extracted and ranked in decreasing order of ROR values, as shown in Table 4. The top five diazepam PTs in terms of number of cases were toxicity to various agents (PT: 10,070,863, n = 2,481), drug abuse (PT: 10,013,654, n = 2,140), completed suicide (PT: 10,010,144, n = 1,012), overdose (PT: 10,033,295, n = 955), and somnolence (PT: 10,041,349, n = 774). The top five PTs in terms of significance were congenital nystagmus (PT: 10,010,562, ROR = 496.173), victim of child abuse (PT: 10,047,401, ROR = 394.682), exposure via father (PT: 10,071,403, ROR = 160.423), poisoning (PT: 10,061,355, ROR = 59.337), and bradypnea (PT: 10,006,102, ROR = 47.099). Notably, several unexpectedly significant AEs were identified that were not labeled in the labeling, including congenital nystagmus (PT: 10,002,959), developmental delay (PT: 10,012,559), pneumonia aspiration (PT: 10,035,669), serotonin syndrome (PT: 10,040,108), pulmonary congestion (PT: 10,037,368), and rhabdomyolysis (PT: 10,039,020). Other unexpected PTs are given in Supplementary Table S1.

**TABLE 4:** Signal strength of diazepam reports at the top 40 preferred term (PT) levels.

System organ class (SOC)	Preferred term (PT)	Case report	ROR (95% CI)	PRR (χ2)	IC (IC025)	EBGM (EBGM05)
Congenital, familial, and genetic disorders	Congenital nystagmus^a^	57	496.17 (360.30–683.29)	495.61 (18,532.9)	5.62 (5.19)	326.79 (237.30)
Social circumstances	Victim of child abuse	54	394.68 (287.44–541.95)	394.26 (14,999.4)	5.53 (5.08)	279.47 (203.53)
Injury, poisoning, and procedural complications	Exposure via father	185	160.42 (137.26–187.49)	159.84 (25,020.9)	6.31 (6.08)	137.10 (117.30)
Injury, poisoning, and procedural complications	Poisoning	609	59.34 (54.65–64.43)	58.63 (32,515.2)	5.67 (5.55)	55.31 (50.94)
Respiratory, thoracic, and mediastinal disorders	Bradypnea	104	57.41 (47.10–69.99)	57.30 (5,427.94)	5.17 (4.88)	54.12 (44.39)
Injury, poisoning, and procedural complications	Exposure via ingestion	134	55.17 (46.35–65.68)	55.03 (6,722.03)	5.24 (4.98)	52.09 (43.76)
Pregnancy, puerperium, and perinatal conditions	Small for gestational age baby	181	43.08 (37.11–50.01)	42.93 (7,093.95)	5.07 (4.86)	41.12 (35.43)
Injury, poisoning, and procedural complications	Poisoning deliberate	220	38.49 (33.63–44.06)	38.33 (7,690.59)	4.99 (4.79)	36.89 (32.23)
Respiratory, thoracic, and mediastinal disorders	Respiratory depression	357	35.07 (31.55–38.99)	34.83 (11,321.2)	4.95 (4.79)	33.64 (30.26)
Psychiatric disorders	Drug abuse	2,140	32.48 (31.08–33.94)	31.15 (60,560.0)	4.90 (4.83)	30.20 (28.90)
Eye disorders	Miosis	174	29.02 (24.95–33.76)	28.93 (4,554.30)	4.60 (4.38)	28.11 (24.17)
Psychiatric disorders	Sopor	319	27.08 (24.22–30.28)	26.91 (7,743.72)	4.60 (4.44)	26.21 (23.44)
Respiratory, thoracic, and mediastinal disorders	Respiratory arrest	610	24.43 (22.53–26.49)	24.15 (13,208.3)	4.51 (4.39)	23.58 (21.75)
Nervous system disorders	Nystagmus	122	26.52 (22.15–31.75)	26.46 (2,908.50)	4.42 (4.16)	25.77 (21.53)
General disorders and administration site conditions	Accidental death	45	27.60 (20.52–37.12)	27.57 (1,120.13)	4.10 (3.67)	26.83 (19.94)
Psychiatric disorders	Substance abuse	163	20.97 (17.95–24.50)	20.91 (3,024.41)	4.19 (3.97)	20.48 (17.53)
Eye disorders	Strabismus	55	22.54 (17.25–29.46)	22.52 (1,105.03)	4.00 (3.61)	22.02 (16.85)
Injury, poisoning, and procedural complications	Alcohol poisoning	50	22.69 (17.14–30.03)	22.66 (1,011.52)	3.97 (3.56)	22.16 (16.74)
Nervous system disorders	Coma	725	18.38 (17.07–19.79)	18.13 (11,525.4)	4.12 (4.01)	17.81 (16.54)
Injury, poisoning, and procedural complications	Toxicity to various agents	2,481	17.44 (16.75–18.16)	16.63 (35,940.0)	4.02 (3.96)	16.37 (15.71)
Investigations	Coma scale abnormal	50	17.87 (13.51–23.64)	17.85 (780.82)	3.73 (3.32)	17.54 (13.26)
Psychiatric disorders	Drug use disorder	68	16.82 (13.23–21.38)	16.80 (992.90)	3.75 (3.40)	16.52 (13.00)
Psychiatric disorders	Completed suicide	1,012	13.87 (13.03–14.77)	13.61 (11,676.1)	3.73 (3.64)	13.43 (12.62)
Respiratory, thoracic, and mediastinal disorders	Respiratory acidosis	51	16.58 (12.57–21.88)	16.57 (733.43)	3.65 (3.25)	16.30 (12.36)
Respiratory, thoracic, and mediastinal disorders	Asphyxia	102	14.66 (12.05–17.83)	14.63 (1,275.97)	3.67 (3.39)	14.43 (11.86)
Psychiatric disorders	Alcohol abuse	40	16.38 (11.98–22.39)	16.36 (567.31)	3.56 (3.10)	16.11 (11.78)
Psychiatric disorders	Bradyphrenia	79	13.54 (10.84–16.91)	13.52 (903.03)	3.53 (3.21)	13.34 (10.68)
Psychiatric disorders	Intentional self-injury	244	11.95 (10.53–13.56)	11.90 (2,406.33)	3.49 (3.31)	11.76 (10.36)
Injury, poisoning, and procedural complications	Intentional overdose	580	11.09 (10.21–12.04)	10.97 (5,203.32)	3.42 (3.30)	10.86 (10.00)
Social circumstances	Drug diversion	41	12.56 (9.23–17.10)	12.55 (430.31)	3.29 (2.84)	12.40 (9.11)
Cardiac disorders	Cardio-respiratory arrest	380	10.16 (9.18–11.25)	10.09 (3,083.13)	3.29 (3.14)	10.00 (9.03)
Nervous system disorders	Depressed level of consciousness	318	9.56 (8.56–10.68)	9.51 (2,398.11)	3.20 (3.04)	9.42 (8.43)
Nervous system disorders	Sedation	191	9.68 (8.40–11.17)	9.65 (1,466.99)	3.19 (2.99)	9.56 (8.29)
Nervous system disorders	Altered state of consciousness	163	9.80 (8.39–11.43)	9.77 (1,270.27)	3.20 (2.97)	9.68 (8.29)
General disorders and administration site conditions	Potentiating drug interaction	33	11.60 (8.23–16.36)	11.60 (315.69)	3.13 (2.63)	11.47 (8.14)
Nervous system disorders	Hypoxic-ischemic encephalopathy	38	11.33 (8.23–15.60)	11.32 (353.32)	3.15 (2.68)	11.20 (8.13)
Surgical and medical procedures	Self-medication	38	11.17 (8.11–15.38)	11.16 (347.42)	3.13 (2.67)	11.04 (8.02)
General disorders and administration site conditions	Developmental delay^a^	77	10.00 (7.99–12.52)	9.99 (616.60)	3.15 (2.82)	9.90 (7.91)
Cardiac disorders	Cardiac arrest	605	8.53 (7.87–9.25)	8.44 (3,941.38)	3.05 (2.93)	8.38 (7.73)
General disorders and administration site conditions	Hypothermia	81	9.61 (7.72–11.96)	9.60 (617.57)	3.11 (2.79)	9.51 (7.64)

^a^ indicates statistically significant signals in the algorithm; ROR, reporting odds ratio; CI, confidence interval; PRR, proportional reporting ratio; χ2, chi-square; IC, information component; IC025, the lower limit of 95% CI of the IC; EBGM, empirical Bayesian geometric mean; EBGM05, the lower limit of 95% CI of EBGM.

## Discussion

Generally, the majority of efficacy and safety data for drugs originate from preclinical and clinical trials (Kim et al., 2018). However, factors such as trial design and relatively small sample sizes can make it challenging to fully elucidate the effects of drugs on humans in the real world, particularly regarding safety. Therefore, focusing on the risk signals of adverse drug effects in clinical applications becomes crucial for evaluating the safety of drugs and achieving a balance between benefits and risks in clinical decision-making. In this study, we collected and evaluated the safety of diazepam through pharmacovigilance, using a large sample of real-world data. The aim is to provide a reference for medication safety in clinical practice.

A total of 15,546 AE reports were collected from the FAERS database, originating from different countries and regions around the world for the years 2004 to 2023. The incidence of diazepam-related AEs was slightly higher in women (44.19%) than in men (42.99%), which may be related to the antagonistic effect of progesterone and its metabolites on diazepam (Silva et al., 2016), implicitly leading to an increase in the dosage and frequency of diazepam in female patients. The annual trend graph of AE reports from 2004 to Q1 2023 (Supplementary Figure S1) shows that the use of diazepam continued to increase from 2004 to 2019 and then gradually declined. This decline might be attributed to the tightening of clinical sedative drug control and the outbreak of COVID-19, resulting in a strain on medical resources.

According to the disproportionality analysis, the most significant signals at the SOC level were psychiatric disorders, injury, poisoning, and procedural complications; nervous system disorders; respiratory, thoracic, and mediastinal disorders; cardiac disorders; and social circumstances. The significant AEs mainly included poisoning, bradypnea, small for dates baby, respiratory depression, coma, bradyphrenia, altered state of consciousness, cardiac arrest, and suicide attempt. These findings are consistent with AEs reported in the drug insert and clinical safety data (Greenblatt et al., 1978; Finkle et al., 1979). We also identified some new valuable potential unexpected AEs, such as congenital nystagmus, developmental delay, pneumonia aspiration, serotonin syndrome, pulmonary congestion, and rhabdomyolysis. These findings are almost difficult to detect in limited population trials.

Diazepam, like other benzodiazepines, acts as a positive allosteric modulator of the GABAA receptor complex. When it binds to the GABAA receptor, it induces an increase in the inward flow of chloride ions into the neuron. This process leads to hyperpolarization of the postsynaptic membrane, enhancing the neuron’s inhibitory response to GABA and thereby exerting its sedative effects (Nutt and Malizia, 2001). Based on diazepam’s pharmacologic mechanism, psychiatric and various neurologic disorders have become its most common AEs. GABAergic neuronal activity has been reported in regions such as the brainstem, thalamus, hypothalamus, and cerebral cortex, and diazepam has a repressive effect on neurons in these areas as well (Polzin and Barnes, 1976; Fontanesi et al., 2007; Zou et al., 2019). This may be one of the mechanisms underlying fatal AEs, such as respiratory depression and cardiac arrest.

Diazepam and its major metabolites can readily diffuse through the placenta to the fetus after administration due to their high lipid solubility. Subsequently, they bind to plasma proteins of the fetus and accumulate in the liver at high levels, especially if the mother has been treated with diazepam in the first trimester of pregnancy (Erkkola et al., 1974; Mandelli et al., 1975; Kuhnz and Nau, 1983). Previous studies have shown that early pregnancy exposure to diazepam is accompanied by a significantly higher risk of limb malformations, rectal–anal stenosis/atresia, cardiovascular malformations, and a variety of congenital malformations (Czeizel et al., 2003). These risks may be linked to neurotransmitter influences during embryonic development (Zimmerman and Wee 1984). Moreover, the use of benzodiazepines close to term leads to neonatal signs of dependence and withdrawal, including hypotonia, reluctance to suck, apnea, cyanosis, and an impaired metabolic response to cold stress. This is closely related to the pharmacokinetics and placental transfer of benzodiazepines (McElhatton, 1994). It is noteworthy that unreported neonatal AEs such as congenital nystagmus, developmental delay, congenital hypophoria, porphyria, and cryopyrin-associated periodic syndromes were also found in our study; the occurrence of these rare AEs might also be related to the above mechanisms, but the exact mechanism still needs to be further explored. Interestingly, a study has demonstrated that exposure to low-dose diazepam (20 mg/d) during the first trimester of pregnancy does not increase the rate of neonatal complications (Tasci et al., 2009). This can provide medication references for some mothers with severe emesis in pregnancy, but great caution is still needed. Unfortunately, due to factors such as the presence of patients with unknown ages in our dataset and other considerations, we were unable to provide detailed information on the exact number of pregnant users.

The abuse of benzodiazepines has become a growing problem worldwide (Zhang et al., 2018; Maust et al., 2019), and long-term use can lead to diminished efficacy, drug dependence, pharmacogenetic insomnia, and addiction. The 2016 National Drug Abuse Monitoring Annual Report showed that benzodiazepines are among the most abused drugs in China. Meanwhile, our study also found that drug abuse and overdose were among the most reported diazepam PTs, making the clinical control of specific drugs particularly important. It has been shown that due to decreased serum levels of carrier proteins in elderly patients, the half-life and free fraction of the drug in the body increase, allowing the drug to accumulate in the body and making it more susceptible to drug overdose (Calcaterra and Barrow, 2014). In populations with tobacco or e-cigarette use, exposure to nicotine impairs the GABAA receptor function in the ventral tegmental area, resulting in altered effects of diazepam on dopamine (DA) circuits and increased consumption of diazepam (Ostroumov et al., 2020). For individuals with a history of moderate alcohol consumption, a family history of alcoholism, and greater anxiety, benzodiazepines have more potent pharmacologic effects, undoubtedly exacerbating the risk of overdose and addiction (Juergens, 1991; Wang et al., 2023). Fortunately, the number of reports of diazepam-related AEs has decreased each year since 2019, which may represent a positive trend.

The GABAA receptor family comprises four subunits (α1, α2, α3, and α5), and benzodiazepines exert their effects by binding to these subunits (Atack, 2005). Among them, the α2 and α3 subunits mediate muscle relaxation and anxiolytic effects (Mohler et al., 2002). A study indicated that zolpidem impairs oral coordination, pharyngeal contraction strength, pharyngeal clearance rate, throat protection, and spontaneous swallowing frequency in healthy adults, thereby increasing the risk of aspiration pneumonia (Hardemark Cedborg et al., 2015). Compared to other benzodiazepines, diazepam has a higher affinity for peripheral benzodiazepine receptors, thus posing a higher risk of aspiration pneumonia (Cheng et al., 2018). Additionally, benzodiazepines may increase the risk of pneumonia by acting on peripheral receptors and inhibiting immune regulation and cell responses (Wei et al., 2010). However, the exact relationship between diazepam and the risk of pneumonia requires further investigation.

Although we were unable to uncover the detailed mechanisms linking diazepam to certain adverse events (e.g., rhabdomyolysis and serotonin syndrome) in the current study, clinical case reports of such events abound (Dagtekin et al., 2011). A cross-sectional survey on dystonia in children indicated rhabdomyolysis-related dystonia in some children administered with oral diazepam (Saini et al., 2022). Furthermore, an animal study demonstrated a notable increase in the solubility of serum M1 and M5 in rats following diazepam use (Lagard et al., 2016). These findings suggest a potential association between these adverse events and diazepam use. Thus, there is a need for more sophisticated studies to unveil the exact pathogenesis of these rare yet potentially serious adverse events, offering a more comprehensive safety assessment for clinical practice.

The study also has some limitations. First, FAERS relies on spontaneous reporting, introducing a reporting bias that can lead to underestimation or underreporting of AEs, affecting the accuracy of the data. This limitation is particularly significant in age groups, as the occurrence of certain AEs is closely associated with age. Second, incomplete data, missing key information, and insufficient total number of patients using diazepam made it impossible to calculate the true incidence of each AE. Population heterogeneity further complicates matters, as study participants encompass a diverse range of ages, genders, races, and health conditions. Additionally, the presence of time delays and confounding factors makes the timely identification and assessment of new safety signals more challenging. Lastly, variations in healthcare levels may affect the consistency of reporting and evaluating drug safety. Our study serves as a clinical warning and supplements the rare AE system in FAERS. It cannot explain the detailed pathogenesis, and large-scale prospective or retrospective studies are needed to validate our results and elucidate the exact mechanism.

## Conclusion

We conducted a pharmacovigilance analysis based on real data from the FAERS database using the disproportionality method to unveil safety signals and potential risks associated with diazepam use. The AEs uncovered in this study were generally consistent with those specified, while some unexpectedly significant AEs, such as congenital nystagmus, developmental delay, serotonin syndrome, and rhabdomyolysis, were also detected. The finding of these strong signaling AEs, to some extent, supplements the relatively small sample size of clinical studies on this drug. However, further prospective clinical studies are needed to confirm and elucidate the association between diazepam and these AEs, considering the presence of reporting bias, data incompleteness, population heterogeneity, and other confounding factors that may limit the results of data analysis. The results of this study can contribute to supplementing the FAERS lineage system for rare AEs, providing a novel and unique perspective on the discovery of such events.

## Data Availability

The original contributions presented in the study are included in the article/Supplementary Material; further inquiries can be directed to the corresponding author.
