# Supplementary material for: A real-world pharmacovigilance study of FDA adverse event reporting system events for diazepam
Source: Front Pharmacol. 2024 Jan 24;15:1278442. doi: 10.3389/fphar.2024.1278442 (PMC10847318; doi:10.3389/fphar.2024.1278442)
Supplement: Supplementary file 1 [file Table1.DOCX]

Supplement Table1: Signal strength of reports of diazepam at the Preferred Term (PT) level

| **SYSTEM ORGAN CLASS（SOC）** | **PREFERRED TERMS（PT）** | **Case Report** | **ROR(95％ CI)** | **PRR(χ2)** | **IC(IC025)** | **EBGM(EBGM05)** |
| --- | --- | --- | --- | --- | --- | --- |
| Injury, poisoning and procedural complications | Toxicity to various agents | 2481 | 17.44（16.75-18.16） | 16.63(35940.05) | 4.03（3.96） | 16.37(15.71) |
| Psychiatric disorders | Drug abuse | 2140 | 32.48（31.08-33.94） | 31.15(60560.05) | 4.92（4.83） | 30.2(28.9) |
| Psychiatric disorders | Completed suicide | 1012 | 13.87（13.03-14.77） | 13.61(11676.09) | 3.75（3.64） | 13.43(12.62) |
| Injury, poisoning and procedural complications | Overdose | 955 | 5.23（4.9-5.57） | 5.15(3184.47) | 2.36（2.26） | 5.12(4.8) |
| Nervous system disorders | Somnolence | 774 | 4.67（4.35-5.02） | 4.61(2188.42) | 2.2（2.09） | 4.6(4.28) |
| Nervous system disorders | Coma | 725 | 18.38（17.07-19.79） | 18.13(11525.37) | 4.15（4.01） | 17.81(16.54) |
| Respiratory, thoracic and mediastinal disorders | Respiratory arrest | 610 | 24.43（22.53-26.49） | 24.15(13208.27) | 4.56（4.39） | 23.58(21.75) |
| Injury, poisoning and procedural complications | Poisoning | 609 | 59.34（54.65-64.43） | 58.63(32515.16) | 5.79（5.55） | 55.31(50.94) |
| Cardiac disorders | Cardiac arrest | 605 | 8.53（7.87-9.25） | 8.44(3941.38) | 3.07（2.93） | 8.38(7.73) |
| Injury, poisoning and procedural complications | Intentional overdose | 580 | 11.09（10.21-12.04） | 10.97(5203.32) | 3.44（3.3） | 10.86(10) |
| General disorders and administration site conditions | Drug interaction | 570 | 4.31（3.97-4.68） | 4.27(1427.49) | 2.09（1.96） | 4.26(3.92) |
| Injury, poisoning and procedural complications | Intentional product misuse | 499 | 5.31（4.86-5.8） | 5.26(1716.9) | 2.39（2.25） | 5.24(4.8) |
| Psychiatric disorders | Drug dependence | 487 | 3.11（2.85-3.4） | 3.09(689.73) | 1.63（1.49） | 3.09(2.82) |
| Psychiatric disorders | Suicide attempt | 414 | 8.25（7.49-9.09） | 8.19(2593.98) | 3.02（2.86） | 8.13(7.38) |
| Cardiac disorders | Cardio-respiratory arrest | 380 | 10.16（9.18-11.25） | 10.09(3083.13) | 3.32（3.14） | 10(9.03) |
| Respiratory, thoracic and mediastinal disorders | Respiratory depression | 357 | 35.07（31.55-38.99） | 34.83(11321.25) | 5.07（4.79） | 33.64(30.26) |
| Nervous system disorders | Seizure | 348 | 2.4（2.16-2.66） | 2.39(280.67) | 1.25（1.09） | 2.38(2.15) |
| Nervous system disorders | Loss of consciousness | 327 | 3.01（2.7-3.35） | 2.99(434.03) | 1.58（1.41） | 2.99(2.68) |
| Psychiatric disorders | Sopor | 319 | 27.08（24.22-30.28） | 26.91(7743.72) | 4.71（4.44） | 26.21(23.44) |
| Nervous system disorders | Depressed level of consciousness | 318 | 9.56（8.56-10.68） | 9.51(2398.11) | 3.24（3.04） | 9.42(8.43) |
| Psychiatric disorders | Agitation | 313 | 4.82（4.32-5.39） | 4.8(938.34) | 2.26（2.08） | 4.78(4.28) |
| Psychiatric disorders | Intentional self-injury | 244 | 11.95（10.53-13.56） | 11.9(2406.33) | 3.56（3.31） | 11.76(10.36) |
| General disorders and administration site conditions | Withdrawal syndrome | 231 | 7.07（6.21-8.05） | 7.05(1190.51) | 2.81（2.58） | 7(6.15) |
| Psychiatric disorders | Suicidal ideation | 228 | 2.94（2.58-3.35） | 2.93(289.69) | 1.55（1.35） | 2.93(2.57) |
| Cardiac disorders | Tachycardia | 222 | 3.01（2.64-3.44） | 3.01(296.53) | 1.58（1.38） | 3(2.63) |
| Injury, poisoning and procedural complications | Poisoning deliberate | 220 | 38.49（33.63-44.06） | 38.33(7690.59) | 5.21（4.79） | 36.89(32.23) |
| Psychiatric disorders | Aggression | 205 | 4.64（4.04-5.32） | 4.63(580.18) | 2.2（1.98） | 4.61(4.02) |
| Injury, poisoning and procedural complications | Foetal exposure during pregnancy | 200 | 3.11（2.71-3.58） | 3.11(285.01) | 1.63（1.41） | 3.1(2.7) |
| Nervous system disorders | Sedation | 191 | 9.68（8.4-11.17） | 9.65(1466.99) | 3.26（2.99） | 9.56(8.29) |
| Injury, poisoning and procedural complications | Exposure via father | 185 | 160.42（137.26-187.49） | 159.84(25020.94) | 7.1（6.08） | 137.1(117.3) |
| Pregnancy, puerperium and perinatal conditions | Small for dates baby | 181 | 43.08（37.11-50.01） | 42.93(7093.95) | 5.36（4.86） | 41.12(35.43) |
| Eye disorders | Miosis | 174 | 29.02（24.95-33.76） | 28.93(4554.3) | 4.81（4.38） | 28.11(24.17) |
| Psychiatric disorders | Substance abuse | 163 | 20.97（17.95-24.5） | 20.91(3024.41) | 4.36（3.97） | 20.48(17.53) |
| Nervous system disorders | Altered state of consciousness | 163 | 9.8（8.39-11.43） | 9.77(1270.27) | 3.27（2.97） | 9.68(8.29) |
| Injury, poisoning and procedural complications | Accidental overdose | 145 | 5.03（4.27-5.92） | 5.02(463.93) | 2.32（2.04） | 4.99(4.24) |
| Infections and infestations | Pneumonia aspiration***** | 143 | 7.09（6.02-8.36） | 7.08(740.95) | 2.81（2.51） | 7.03(5.96) |
| Psychiatric disorders | Hallucination | 141 | 2.39（2.02-2.81） | 2.38(112.82) | 1.25（0.99） | 2.38(2.02) |
| Injury, poisoning and procedural complications | Exposure via ingestion | 134 | 55.17（46.35-65.68） | 55.03(6722.03) | 5.7（4.98） | 52.09(43.76) |
| Nervous system disorders | Dysarthria | 133 | 4.13（3.48-4.9） | 4.12(313.39) | 2.04（1.76） | 4.11(3.46) |
| Cardiac disorders | Bradycardia | 130 | 2.82（2.37-3.35） | 2.81(151.41) | 1.49（1.22） | 2.81(2.36) |
| Psychiatric disorders | Delirium | 123 | 4.51（3.77-5.38） | 4.5(333.21) | 2.16（1.86） | 4.48(3.75) |
| Nervous system disorders | Nystagmus | 122 | 26.52（22.15-31.75） | 26.46(2908.5) | 4.69（4.16） | 25.77(21.53) |
| Investigations | Electrocardiogram QT prolonged | 116 | 3.88（3.23-4.66） | 3.87(246.57) | 1.95（1.65） | 3.86(3.22) |
| Investigations | Oxygen saturation decreased | 114 | 2.68（2.23-3.22） | 2.68(119.56) | 1.42（1.13） | 2.67(2.22) |
| Respiratory, thoracic and mediastinal disorders | Pulmonary oedema | 113 | 2.96（2.46-3.57） | 2.96(146.24) | 1.56（1.27） | 2.95(2.45) |
| Musculoskeletal and connective tissue disorders | Rhabdomyolysis***** | 111 | 3.18（2.64-3.84） | 3.18(165.37) | 1.67（1.36） | 3.17(2.63) |
| Psychiatric disorders | Abnormal behaviour | 108 | 2.94（2.43-3.55） | 2.93(137.32) | 1.55（1.25） | 2.93(2.42) |
| Respiratory, thoracic and mediastinal disorders | Bradypnoea | 104 | 57.41（47.1-69.99） | 57.3(5427.94) | 5.76（4.88） | 54.12(44.39) |
| Respiratory, thoracic and mediastinal disorders | Asphyxia | 102 | 14.66（12.05-17.83） | 14.63(1275.97) | 3.85（3.39） | 14.43(11.86) |
| Psychiatric disorders | Restlessness | 100 | 3.17（2.6-3.86） | 3.16(147.66) | 1.66（1.34） | 3.16(2.59) |
| Injury, poisoning and procedural complications | Device use error | 99 | 3.99（3.28-4.86） | 3.99(220.58) | 1.99（1.66） | 3.97(3.26) |
| Psychiatric disorders | Disorientation | 99 | 2.81（2.3-3.42） | 2.8(114.63) | 1.48（1.17） | 2.8(2.3) |
| Nervous system disorders | Unresponsive to stimuli | 96 | 4.41（3.61-5.39） | 4.4(251.23) | 2.13（1.79） | 4.39(3.59) |
| Nervous system disorders | Serotonin syndrome***** | 94 | 6.42（5.24-7.87） | 6.41(426.68) | 2.67（2.3） | 6.38(5.2) |
| Psychiatric disorders | Psychotic disorder | 92 | 3.66（2.98-4.5） | 3.66(177.09) | 1.87（1.53） | 3.65(2.97) |
| Nervous system disorders | Neuroleptic malignant syndrome | 82 | 8.21（6.6-10.2） | 8.19(513.65) | 3.02（2.59） | 8.13(6.54) |
| General disorders and administration site conditions | Hypothermia | 81 | 9.61（7.72-11.96） | 9.6(617.57) | 3.25（2.79） | 9.51(7.64) |
| Respiratory, thoracic and mediastinal disorders | Hypoxia | 81 | 2.86（2.3-3.56） | 2.86(97.53) | 1.51（1.16） | 2.85(2.29) |
| Psychiatric disorders | Panic attack | 81 | 2.63（2.11-3.27） | 2.63(81.4) | 1.39（1.04） | 2.62(2.11) |
| Psychiatric disorders | Bradyphrenia | 79 | 13.54（10.84-16.91） | 13.52(903.03) | 3.74（3.21） | 13.34(10.68) |
| Eye disorders | Mydriasis | 78 | 7.29（5.83-9.11） | 7.28(419.17) | 2.85（2.42） | 7.23(5.78) |
| General disorders and administration site conditions | Developmental delay***** | 77 | 10（7.99-12.52） | 9.99(616.6) | 3.31（2.82） | 9.9(7.91) |
| Nervous system disorders | Encephalopathy | 77 | 3.95（3.15-4.94） | 3.94(168.43) | 1.97（1.59） | 3.93(3.14) |
| Cardiac disorders | Sinus tachycardia | 71 | 5.86（4.64-7.4） | 5.85(283.83) | 2.54（2.11） | 5.82(4.61) |
| Respiratory, thoracic and mediastinal disorders | Respiratory distress | 71 | 3（2.37-3.78） | 2.99(94.04) | 1.58（1.2） | 2.99(2.37) |
| Nervous system disorders | Hypotonia | 69 | 7.88（6.21-9.98） | 7.87(410.22) | 2.97（2.48） | 7.81(6.16) |
| Psychiatric disorders | Drug use disorder | 68 | 16.82（13.23-21.38） | 16.8(992.9) | 4.05（3.4） | 16.52(13) |
| General disorders and administration site conditions | Drug withdrawal syndrome neonatal | 68 | 5.78（4.55-7.34） | 5.77(266.76) | 2.52（2.08） | 5.74(4.52) |
| Investigations | Blood creatine phosphokinase increased***** | 68 | 2.58（2.04-3.28） | 2.58(65.68) | 1.37（0.98） | 2.58(2.03) |
| Respiratory, thoracic and mediastinal disorders | Apnoea | 65 | 9.43（7.38-12.04） | 9.42(484.38) | 3.22（2.69） | 9.34(7.31) |
| Nervous system disorders | Psychomotor hyperactivity | 62 | 4.14（3.23-5.31） | 4.14(146.87) | 2.04（1.61） | 4.12(3.21) |
| Social circumstances | Drug abuser | 59 | 9.27（7.17-11.97） | 9.26(430.34) | 3.2（2.64） | 9.18(7.1) |
| General disorders and administration site conditions | Sudden death | 59 | 6.07（4.7-7.84） | 6.06(247.79) | 2.59（2.1） | 6.03(4.67) |
| Respiratory, thoracic and mediastinal disorders | Aspiration | 58 | 6.68（5.16-8.65） | 6.68(278.07) | 2.73（2.22） | 6.64(5.13) |
| Congenital, familial and genetic disorders | Congenital nystagmus***** | 57 | 496.17（360.3-683.29） | 495.61(18532.87) | 8.35（5.19） | 326.79(237.3) |
| Eye disorders | Strabismus | 55 | 22.54（17.25-29.46） | 22.52(1105.03) | 4.46（3.61） | 22.02(16.85) |
| Social circumstances | Victim of child abuse | 54 | 394.68（287.44-541.95） | 394.26(14999.4) | 8.13（5.08） | 279.47(203.53) |
| Nervous system disorders | Brain oedema | 53 | 5.01（3.82-6.56） | 5(168.96) | 2.32（1.82） | 4.98(3.8) |
| Respiratory, thoracic and mediastinal disorders | Respiratory acidosis | 51 | 16.58（12.57-21.88） | 16.57(733.43) | 4.03（3.25） | 16.3(12.36) |
| Injury, poisoning and procedural complications | Alcohol poisoning | 50 | 22.69（17.14-30.03） | 22.66(1011.52) | 4.47（3.56） | 22.16(16.74) |
| Investigations | Coma scale abnormal | 50 | 17.87（13.51-23.64） | 17.85(780.82) | 4.13（3.32） | 17.54(13.26) |
| Vascular disorders | Circulatory collapse | 47 | 3.1（2.32-4.12） | 3.09(66.38) | 1.63（1.15） | 3.09(2.32) |
| Hepatobiliary disorders | Hepatocellular injury | 47 | 3.04（2.28-4.04） | 3.03(63.92) | 1.6（1.12） | 3.03(2.27) |
| Investigations | Drug screen positive | 46 | 6.63（4.96-8.86） | 6.63(218.21) | 2.72（2.13） | 6.59(4.93) |
| General disorders and administration site conditions | Accidental death | 45 | 27.6（20.52-37.12） | 27.57(1120.13) | 4.75（3.67） | 26.83(19.94) |
| Nervous system disorders | Status epilepticus | 45 | 4.83（3.6-6.47） | 4.83(135.84) | 2.27（1.72） | 4.81(3.59) |
| Vascular disorders | Cyanosis | 43 | 3.17（2.35-4.28） | 3.17(63.65) | 1.66（1.16） | 3.16(2.34) |
| Nervous system disorders | Ataxia | 42 | 4.07（3-5.5） | 4.06(96.58) | 2.02（1.48） | 4.05(2.99) |
| Respiratory, thoracic and mediastinal disorders | Pulmonary congestion***** | 42 | 3.88（2.86-5.25） | 3.87(89.15) | 1.95（1.41） | 3.86(2.85) |
| Social circumstances | Drug diversion | 41 | 12.56（9.23-17.1） | 12.55(430.31) | 3.63（2.84） | 12.4(9.11) |
| Psychiatric disorders | Alcohol abuse | 40 | 16.38（11.98-22.39） | 16.36(567.31) | 4.01（3.1） | 16.11(11.78) |
| Nervous system disorders | Parkinsonism | 39 | 5.06（3.7-6.94） | 5.06(126.37) | 2.33（1.74） | 5.04(3.68) |
| Psychiatric disorders | Dependence | 39 | 4.87（3.55-6.66） | 4.86(119.05) | 2.28（1.69） | 4.84(3.53) |
| Nervous system disorders | Myoclonus | 39 | 3.88（2.83-5.32） | 3.88(83.05) | 1.95（1.39） | 3.87(2.82) |
| Nervous system disorders | Akathisia | 39 | 3.36（2.46-4.61） | 3.36(64.47) | 1.75（1.21） | 3.35(2.45) |
| Nervous system disorders | Hypoxic-ischaemic encephalopathy | 38 | 11.33（8.23-15.6） | 11.32(353.32) | 3.49（2.68） | 11.2(8.13) |
| Surgical and medical procedures | Self-medication | 38 | 11.17（8.11-15.38） | 11.16(347.42) | 3.46（2.67） | 11.04(8.02) |
| Respiratory, thoracic and mediastinal disorders | Obstructive airways disorder | 36 | 4.01（2.89-5.56） | 4(80.8) | 2（1.41） | 3.99(2.88) |
| Psychiatric disorders | Catatonia | 34 | 8.31（5.93-11.64） | 8.3(216.51) | 3.04（2.28） | 8.24(5.88) |
| Nervous system disorders | Drug withdrawal convulsions | 33 | 21.41（15.16-30.24） | 21.4(627.67) | 4.39（3.22） | 20.95(14.84) |
| General disorders and administration site conditions | Potentiating drug interaction | 33 | 11.6（8.23-16.36） | 11.6(315.69) | 3.52（2.63） | 11.47(8.14) |
| Psychiatric disorders | Logorrhoea | 33 | 10.43（7.4-14.7） | 10.42(278.13) | 3.37（2.52） | 10.32(7.32) |
| Musculoskeletal and connective tissue disorders | Muscle rigidity | 33 | 3.31（2.35-4.67） | 3.31(53.11) | 1.72（1.13） | 3.3(2.35) |
| Psychiatric disorders | Schizophrenia | 33 | 3.3（2.34-4.64） | 3.3(52.63) | 1.72（1.13） | 3.29(2.34) |
| Respiratory, thoracic and mediastinal disorders | Hypoventilation | 32 | 10.8（7.62-15.31） | 10.8(281.28) | 3.42（2.54） | 10.69(7.54) |
| Gastrointestinal disorders | Salivary hypersecretion | 32 | 3.91（2.76-5.53） | 3.91(68.98) | 1.96（1.34） | 3.9(2.75) |
| Surgical and medical procedures | Drug detoxification | 31 | 43.89（30.62-62.91） | 43.86(1241.48) | 5.39（3.68） | 41.98(29.29) |
| Injury, poisoning and procedural complications | Maternal drugs affecting foetus | 31 | 3.69（2.59-5.25） | 3.69(60.57) | 1.88（1.25） | 3.68(2.59) |
| Psychiatric disorders | Agoraphobia | 30 | 24.48（17.03-35.17） | 24.46(658.26) | 4.58（3.25） | 23.88(16.62) |
| General disorders and administration site conditions | Alcohol interaction | 30 | 17.68（12.32-25.37） | 17.67(463.21) | 4.12（2.98） | 17.37(12.1) |
| Injury, poisoning and procedural complications | Maternal exposure timing unspecified | 29 | 11.16（7.74-16.1） | 11.16(265.08) | 3.46（2.52） | 11.04(7.65) |
| Nervous system disorders | Brain injury | 29 | 3.32（2.31-4.78） | 3.32(46.89) | 1.73（1.09） | 3.31(2.3) |
| Social circumstances | Victim of chemical submission | 28 | 120.7（81.46-178.84） | 120.63(2949.92) | 6.74（3.96） | 107.23(72.37) |
| Social circumstances | Housebound | 28 | 94.35（63.99-139.12） | 94.3(2352.7) | 6.43（3.89） | 85.92(58.27) |
| Congenital, familial and genetic disorders | Microcephaly | 28 | 26.19（17.99-38.13） | 26.18(660.02) | 4.67（3.25） | 25.51(17.52) |
| Nervous system disorders | Bradykinesia | 28 | 6.91（4.76-10.02） | 6.91(140.46) | 2.78（1.98） | 6.87(4.73) |
| Cardiac disorders | Sinus bradycardia | 28 | 3.36（2.32-4.88） | 3.36(46.32) | 1.75（1.1） | 3.35(2.31) |
| Nervous system disorders | Psychomotor skills impaired | 26 | 11.52（7.82-16.96） | 11.51(246.63) | 3.51（2.48） | 11.39(7.73) |
| Musculoskeletal and connective tissue disorders | Posture abnormal | 26 | 9.34（6.35-13.75） | 9.34(191.72) | 3.21（2.27） | 9.26(6.29) |
| Injury, poisoning and procedural complications | Prescription drug used without a prescription | 26 | 6.81（4.63-10.02） | 6.81(127.96) | 2.76（1.92） | 6.77(4.6) |
| Social circumstances | Homicide | 25 | 16.97（11.42-25.2） | 16.96(368.9) | 4.06（2.81） | 16.68(11.23) |
| Psychiatric disorders | Post-traumatic stress disorder | 25 | 4.75（3.21-7.04） | 4.75(73.68) | 2.24（1.48） | 4.73(3.19) |
| General disorders and administration site conditions | Paradoxical drug reaction | 24 | 8.45（5.65-12.62） | 8.44(156.09) | 3.07（2.11） | 8.38(5.6) |
| Nervous system disorders | Incoherent | 24 | 4.12（2.76-6.16） | 4.12(56.49) | 2.04（1.29） | 4.11(2.75) |
| Pregnancy, puerperium and perinatal conditions | Foetal death | 23 | 3.94（2.62-5.94） | 3.94(50.29) | 1.97（1.22） | 3.93(2.61) |
| Cardiac disorders | Arteriosclerosis coronary artery***** | 23 | 3.76（2.49-5.66） | 3.75(46.29) | 1.9（1.16） | 3.74(2.49) |
| General disorders and administration site conditions | Hyperthermia | 23 | 3.54（2.35-5.33） | 3.54(41.66) | 1.82（1.08） | 3.53(2.34) |
| Investigations | Electrocardiogram QRS complex prolonged | 22 | 5.55（3.65-8.43） | 5.54(81.48) | 2.46（1.6） | 5.52(3.63) |
| Vascular disorders | Visceral congestion***** | 21 | 58.41（37.59-90.76） | 58.39(1116.36) | 5.78（3.36） | 55.09(35.45) |
| Social circumstances | Alcohol use | 21 | 5.86（3.81-8.99） | 5.85(84.02) | 2.54（1.64） | 5.82(3.79) |
| Congenital, familial and genetic disorders | Optic nerve hypoplasia | 20 | 294.41（178.34-486.01） | 294.29(4470.41) | 7.82（3.57） | 225.28(136.47) |
| Injury, poisoning and procedural complications | Sedation complication | 20 | 12.92（8.31-20.09） | 12.92(216.95) | 3.67（2.4） | 12.76(8.21) |
| Psychiatric disorders | Psychomotor retardation | 20 | 8.69（5.59-13.5） | 8.69(134.83) | 3.11（2.03） | 8.62(5.55) |
| Nervous system disorders | Toxic encephalopathy | 20 | 5.24（3.38-8.14） | 5.24(68.29) | 2.38（1.49） | 5.22(3.36) |
| General disorders and administration site conditions | Drug tolerance | 20 | 4.78（3.08-7.43） | 4.78(59.56) | 2.25（1.38） | 4.76(3.07) |
| Immune system disorders | Multiple allergies | 20 | 3.35（2.16-5.2） | 3.35(32.86) | 1.74（0.96） | 3.34(2.15) |
| Musculoskeletal and connective tissue disorders | Hypotonia neonatal | 19 | 13.8（8.78-21.71） | 13.8(222.35) | 3.77（2.41） | 13.62(8.66) |
| Investigations | Blood bicarbonate decreased | 19 | 11.28（7.17-17.73） | 11.27(175.81) | 3.48（2.24） | 11.15(7.09) |
| Nervous system disorders | Stupor | 19 | 6.52（4.15-10.24） | 6.52(88.12) | 2.7（1.7） | 6.48(4.13) |
| Respiratory, thoracic and mediastinal disorders | Snoring | 19 | 6.01（3.83-9.44） | 6.01(78.85) | 2.58（1.61） | 5.98(3.81) |
| Psychiatric disorders | Behaviour disorder | 19 | 5.52（3.51-8.66） | 5.52(69.84) | 2.46（1.52） | 5.49(3.5) |
| Injury, poisoning and procedural complications | Intercepted medication error | 19 | 4.33（2.76-6.8） | 4.33(48.44) | 2.11（1.24） | 4.31(2.75) |
| Investigations | Blood pH decreased | 18 | 10.81（6.79-17.21） | 10.81(158.41) | 3.42（2.16） | 10.7(6.72) |
| Psychiatric disorders | Depersonalisation/derealisation disorder | 18 | 9.36（5.89-14.9） | 9.36(133.13) | 3.21（2.03） | 9.28(5.83) |
| Investigations | Antipsychotic drug level increased | 18 | 7.64（4.81-12.16） | 7.64(103.08) | 2.92（1.83） | 7.59(4.77) |
| Respiratory, thoracic and mediastinal disorders | Hypercapnia | 18 | 7.38（4.64-11.73） | 7.37(98.4) | 2.87（1.79） | 7.32(4.61) |
| Nervous system disorders | Hyperreflexia | 18 | 5.78（3.64-9.19） | 5.78(70.72) | 2.52（1.54） | 5.75(3.62) |
| Cardiac disorders | Left ventricular hypertrophy | 18 | 4.79（3.02-7.62） | 4.79(53.74) | 2.25（1.33） | 4.77(3) |
| Psychiatric disorders | Social avoidant behaviour | 18 | 3.3（2.08-5.24） | 3.3(28.76) | 1.72（0.89） | 3.29(2.07) |
| Cardiac disorders | Left ventricular dysfunction | 18 | 3.26（2.05-5.18） | 3.26(28.14) | 1.7（0.88） | 3.25(2.05) |
| Congenital, familial and genetic disorders | Strabismus congenital | 17 | 133.32（80.26-221.46） | 133.28(1958.87) | 6.87（3.25） | 117.1(70.49) |
| Investigations | Vital capacity decreased | 17 | 47.98（29.48-78.1） | 47.96(744.41) | 5.51（3.02） | 45.72(28.09) |
| Respiratory, thoracic and mediastinal disorders | Reversible airways obstruction | 17 | 40.87（25.15-66.41） | 40.85(633.86) | 5.29（2.95） | 39.22(24.14) |
| Investigations | Sputum culture positive | 17 | 21.34（13.2-34.52） | 21.34(322.36) | 4.39（2.62） | 20.89(12.92) |
| Respiratory, thoracic and mediastinal disorders | Bronchial secretion retention | 17 | 17.95（11.11-29.01） | 17.95(267.03) | 4.14（2.51） | 17.63(10.91) |
| Investigations | Apgar score low | 17 | 14.39（8.92-23.24） | 14.39(208.66) | 3.83（2.35） | 14.19(8.79) |
| Social circumstances | Physical assault | 17 | 9.37（5.81-15.11） | 9.37(125.81) | 3.21（1.98） | 9.29(5.76) |
| Product issues | Suspected counterfeit product | 17 | 6.25（3.88-10.06） | 6.24(74.39) | 2.63（1.58） | 6.21(3.85) |
| Investigations | Respiratory rate decreased | 17 | 6.2（3.85-9.99） | 6.2(73.61) | 2.62（1.58） | 6.16(3.83) |
| Hepatobiliary disorders | Jaundice cholestatic | 17 | 5.53（3.43-8.91） | 5.53(62.73) | 2.46（1.46） | 5.5(3.42) |
| Psychiatric disorders | Suicidal behaviour | 17 | 4.59（2.85-7.38） | 4.58(47.41) | 2.19（1.25） | 4.57(2.84) |
| Nervous system disorders | Judgement impaired | 16 | 11.74（7.17-19.22） | 11.74(155.23) | 3.54（2.13） | 11.61(7.09) |
| Product issues | Product counterfeit | 16 | 11.21（6.85-18.36） | 11.21(147.09) | 3.47（2.09） | 11.09(6.78) |
| Investigations | Oxygen saturation abnormal | 16 | 7.97（4.87-13.04） | 7.97(96.73) | 2.98（1.79） | 7.91(4.84) |
| General disorders and administration site conditions | Brain death | 16 | 6.07（3.72-9.93） | 6.07(67.37) | 2.59（1.52） | 6.04(3.69) |
| Psychiatric disorders | Panic disorder | 16 | 4.68（2.86-7.65） | 4.68(46.03) | 2.22（1.24） | 4.66(2.85) |
| Immune system disorders | Reaction to excipient | 16 | 3.9（2.38-6.37） | 3.9(34.3) | 1.96（1.03） | 3.88(2.38) |
| Congenital, familial and genetic disorders | Cleft palate | 15 | 8.77（5.27-14.58） | 8.76(102.24) | 3.12（1.83） | 8.69(5.23) |
| Psychiatric disorders | Selective eating disorder | 15 | 7.22（4.35-12） | 7.22(79.78) | 2.84（1.65） | 7.17(4.32) |
| Musculoskeletal and connective tissue disorders | Compartment syndrome | 15 | 6.56（3.95-10.91） | 6.56(70.25) | 2.71（1.55） | 6.53(3.93) |
| Congenital, familial and genetic disorders | Dysmorphism | 15 | 6.01（3.62-9.99） | 6.01(62.3) | 2.58（1.47） | 5.98(3.6) |
| Hepatobiliary disorders | Hepatic necrosis | 15 | 4.38（2.64-7.27） | 4.38(38.92) | 2.13（1.13） | 4.36(2.63) |
| Cardiac disorders | Pulseless electrical activity | 15 | 3.57（2.15-5.93） | 3.57(27.63) | 1.83（0.89） | 3.56(2.14) |
| Social circumstances | Sexual abuse | 14 | 41.86（24.51-71.48） | 41.84(534.77) | 5.33（2.71） | 40.13(23.5) |
| Respiratory, thoracic and mediastinal disorders | Neonatal asphyxia | 14 | 23.54（13.85-40） | 23.53(294.81) | 4.52（2.47） | 22.99(13.53) |
| Investigations | PCO2 increased | 14 | 21.67（12.76-36.81） | 21.67(269.88) | 4.41（2.42） | 21.21(12.49) |
| Investigations | Blood alcohol increased | 14 | 18.2（10.72-30.88） | 18.19(223.23) | 4.16（2.32） | 17.87(10.53) |
| Psychiatric disorders | Learning disorder | 14 | 12.03（7.1-20.39） | 12.03(139.84) | 3.57（2.03） | 11.89(7.02) |
| Endocrine disorders | Diabetes insipidus***** | 14 | 7.27（4.3-12.3） | 7.27(75.08) | 2.85（1.6） | 7.22(4.27) |
| Psychiatric disorders | Hypomania | 14 | 4.36（2.58-7.38） | 4.36(36.11) | 2.12（1.08） | 4.35(2.57) |
| Psychiatric disorders | Impulsive behaviour | 14 | 4.33（2.56-7.31） | 4.33(35.63) | 2.11（1.07） | 4.31(2.55) |
| Immune system disorders | Rhesus incompatibility | 13 | 161.52（89.73-290.73） | 161.48(1773.83) | 7.11（2.85） | 138.3(76.83) |
| Injury, poisoning and procedural complications | Accidental poisoning | 13 | 16.99（9.82-29.4） | 16.99(192.18) | 4.06（2.2） | 16.71(9.65) |
| Respiratory, thoracic and mediastinal disorders | Infantile apnoea | 13 | 10.59（6.13-18.3） | 10.59(111.68) | 3.39（1.87） | 10.49(6.07) |
| Psychiatric disorders | Disinhibition | 13 | 9.13（5.29-15.77） | 9.13(93.22) | 3.18（1.75） | 9.05(5.24) |
| Nervous system disorders | Slow speech | 13 | 7.3（4.23-12.6） | 7.3(70.11) | 2.86（1.55） | 7.25(4.2) |
| Surgical and medical procedures | Endotracheal intubation | 13 | 5.28（3.06-9.11） | 5.28(44.85) | 2.39（1.24） | 5.26(3.05) |
| Investigations | Electroencephalogram abnormal | 13 | 4.52（2.62-7.8） | 4.52(35.49) | 2.17（1.08） | 4.5(2.61) |
| Psychiatric disorders | Anxiety disorder | 13 | 4.49（2.61-7.75） | 4.49(35.13) | 2.16（1.07） | 4.48(2.6) |
| Psychiatric disorders | Self-injurious ideation | 13 | 3.75（2.18-6.47） | 3.75(26.15) | 1.9（0.87） | 3.74(2.17) |
| Social circumstances | Victim of sexual abuse | 12 | 27.33（15.4-48.52） | 27.33(295.91) | 4.73（2.35） | 26.6(14.98) |
| Psychiatric disorders | Substance use disorder | 12 | 13.49（7.63-23.85） | 13.49(136.81) | 3.73（1.97） | 13.31(7.53) |
| Injury, poisoning and procedural complications | Brain herniation | 12 | 4.74（2.69-8.36） | 4.74(35.25) | 2.24（1.07） | 4.72(2.68) |
| Renal and urinary disorders | Renal ischaemia | 11 | 27.33（15.01-49.77） | 27.33(271.25) | 4.73（2.24） | 26.6(14.6) |
| Investigations | PO2 increased | 11 | 23.08（12.69-41.97） | 23.07(226.8) | 4.5（2.17） | 22.55(12.4) |
| Congenital, familial and genetic disorders | Syndactyly | 11 | 15.1（8.32-27.39） | 15.09(142.52) | 3.89（1.95） | 14.88(8.2) |
| General disorders and administration site conditions | Drowning | 11 | 12.77（7.04-23.15） | 12.77(117.74) | 3.66（1.84） | 12.61(6.96) |
| Psychiatric disorders | Psychotic behaviour | 11 | 12.39（6.84-22.47） | 12.39(113.73) | 3.61（1.82） | 12.25(6.76) |
| Psychiatric disorders | Personality disorder | 11 | 4.91（2.71-8.87） | 4.9(34.02) | 2.29（1.05） | 4.88(2.7) |
| Nervous system disorders | Clonus | 11 | 4.74（2.62-8.58） | 4.74(32.34) | 2.24（1.01） | 4.72(2.61) |
| Nervous system disorders | Areflexia | 11 | 4.68（2.59-8.47） | 4.68(31.68) | 2.22（1） | 4.66(2.58) |
| Nervous system disorders | Clumsiness | 11 | 4.6（2.54-8.31） | 4.6(30.81) | 2.2（0.98） | 4.58(2.53) |
| Psychiatric disorders | Depressive symptom | 11 | 4.06（2.25-7.34） | 4.06(25.27) | 2.02（0.86） | 4.05(2.24) |
| Congenital, familial and genetic disorders | Congenital visual acuity reduced | 10 | 318.88（155.88-652.33） | 318.82(2376.15) | 7.9（2.42） | 239.36(117.01) |
| Social circumstances | Chemical submission | 10 | 88.58（46.34-169.32） | 88.56(792.35) | 6.34（2.38） | 81.14(42.45) |
| Investigations | Blood osmolarity increased | 10 | 37.96（20.18-71.42） | 37.95(346.08) | 5.19（2.22） | 36.54(19.42) |
| Pregnancy, puerperium and perinatal conditions | Breech presentation | 10 | 8.56（4.59-15.95） | 8.56(66.13) | 3.09（1.46） | 8.49(4.55) |
| Psychiatric disorders | Illusion | 10 | 7.16（3.84-13.34） | 7.16(52.6) | 2.83（1.32） | 7.11(3.82) |
| Psychiatric disorders | Derealisation | 10 | 5.95（3.2-11.09） | 5.95(40.94) | 2.57（1.16） | 5.92(3.18) |
| Pregnancy, puerperium and perinatal conditions | Foetal distress syndrome | 10 | 5.33（2.86-9.93） | 5.33(35) | 2.41（1.06） | 5.31(2.85) |
| Investigations | Toxicologic test abnormal | 9 | 14.84（7.68-28.68） | 14.84(114.41) | 3.87（1.71） | 14.63(7.57) |
| Nervous system disorders | Psychogenic seizure | 9 | 12.11（6.27-23.37） | 12.11(90.56) | 3.58（1.59） | 11.97(6.2) |
| Psychiatric disorders | Alcohol withdrawal syndrome | 9 | 12.07（6.26-23.3） | 12.07(90.26) | 3.58（1.59） | 11.93(6.18) |
| Cardiac disorders | Bradycardia neonatal | 9 | 8.32（4.32-16.03） | 8.32(57.44) | 3.05（1.34） | 8.25(4.28) |
| Congenital, familial and genetic disorders | Hypertrophic cardiomyopathy | 9 | 7.41（3.85-14.28） | 7.41(49.5) | 2.88（1.25） | 7.36(3.82) |
| Psychiatric disorders | Substance-induced psychotic disorder | 9 | 5.93（3.08-11.42） | 5.93(36.65) | 2.56（1.07） | 5.9(3.06) |
| Eye disorders | Pupil fixed | 9 | 5.62（2.92-10.83） | 5.62(34) | 2.48（1.02） | 5.6(2.91) |
| Nervous system disorders | Autonomic nervous system imbalance | 9 | 4.05（2.1-7.79） | 4.05(20.58) | 2.01（0.72） | 4.04(2.1) |
| Nervous system disorders | Hypercapnic coma | 8 | 75.03（36.53-154.09） | 75.02(541.74) | 6.12（2.01） | 69.63(33.9) |
| Psychiatric disorders | Delirium tremens | 8 | 21.32（10.58-42.96） | 21.31(151.51) | 4.38（1.73） | 20.87(10.36) |
| Investigations | Opiates positive | 8 | 17.39（8.64-35） | 17.39(121.37) | 4.1（1.64） | 17.1(8.5) |
| Social circumstances | Victim of crime | 8 | 14.55（7.24-29.25） | 14.55(99.41) | 3.84（1.56） | 14.34(7.14) |
| Congenital, familial and genetic disorders | Cleft lip | 8 | 9.59（4.78-19.24） | 9.59(60.93) | 3.25（1.32） | 9.5(4.74) |
| Renal and urinary disorders | Bladder dilatation | 8 | 7.6（3.79-15.24） | 7.6(45.48) | 2.92（1.16） | 7.55(3.76) |
| Nervous system disorders | Slow response to stimuli | 8 | 7.57（3.77-15.18） | 7.57(45.25) | 2.91（1.16） | 7.52(3.75) |
| Injury, poisoning and procedural complications | Vasoplegia syndrome | 8 | 7.55（3.77-15.15） | 7.55(45.13) | 2.91（1.16） | 7.5(3.74) |
| Investigations | Mean cell volume decreased | 8 | 6.35（3.17-12.73） | 6.35(35.82) | 2.66（1.02） | 6.31(3.15) |
| Eye disorders | Pupillary reflex impaired | 8 | 5.14（2.57-10.3） | 5.14(26.53) | 2.36（0.85） | 5.12(2.55) |
| Congenital, familial and genetic disorders | Hypospadias | 8 | 4.34（2.17-8.7） | 4.34(20.49) | 2.11（0.69） | 4.33(2.16) |
| Nervous system disorders | Delayed visual maturation | 7 | 956.58（335.51-2727.3） | 956.45(3340.58) | 8.9（1.71） | 478.72(167.91) |
| Psychiatric disorders | Delusion of replacement | 7 | 94.31（43.38-205.02） | 94.3(588.17) | 6.43（1.81） | 85.92(39.53) |
| Investigations | Postmortem blood drug level abnormal | 7 | 66.3（30.82-142.63） | 66.29(420.95) | 5.96（1.79） | 62.06(28.85) |
| Psychiatric disorders | Withdrawal catatonia | 7 | 62.58（29.13-134.44） | 62.57(398.07) | 5.88（1.78） | 58.79(27.37) |
| Surgical and medical procedures | Multiple drug therapy | 7 | 17.39（8.24-36.73） | 17.39(106.2) | 4.1（1.47） | 17.1(8.1) |
| Congenital, familial and genetic disorders | Atrioventricular septal defect | 7 | 16.57（7.85-34.99） | 16.57(100.68) | 4.03（1.45） | 16.31(7.72) |
| Respiratory, thoracic and mediastinal disorders | Irregular breathing | 7 | 15.61（7.4-32.94） | 15.61(94.16) | 3.94（1.43） | 15.37(7.28) |
| Nervous system disorders | Anterograde amnesia | 7 | 13.95（6.61-29.42） | 13.95(82.93) | 3.78（1.38） | 13.76(6.53) |
| Psychiatric disorders | Persistent depressive disorder | 7 | 11.09（5.26-23.36） | 11.08(63.49) | 3.46（1.26） | 10.97(5.21) |
| Cardiac disorders | Bradycardia foetal | 7 | 10.24（4.86-21.56） | 10.24(57.73) | 3.34（1.22） | 10.14(4.81) |
| Nervous system disorders | Opisthotonus | 7 | 9.47（4.5-19.94） | 9.47(52.51) | 3.23（1.17） | 9.39(4.46) |
| Nervous system disorders | Cerebellar ataxia | 7 | 8.21（3.9-17.27） | 8.2(43.91) | 3.03（1.08） | 8.14(3.87) |
| Social circumstances | Substance use | 7 | 8.01（3.81-16.85） | 8.01(42.58) | 2.99（1.06） | 7.95(3.78) |
| Congenital, familial and genetic disorders | Congenital central nervous system anomaly | 7 | 7.5（3.56-15.78） | 7.5(39.11) | 2.9（1.02） | 7.45(3.54) |
| Psychiatric disorders | Phobia | 7 | 6.44（3.06-13.55） | 6.44(31.98) | 2.68（0.91） | 6.41(3.05) |
| Investigations | Anion gap increased | 7 | 6.15（2.92-12.93） | 6.15(29.98) | 2.61（0.87） | 6.12(2.91) |
| Nervous system disorders | Anticholinergic syndrome | 7 | 6.07（2.88-12.75） | 6.06(29.42) | 2.59（0.86） | 6.03(2.87) |
| Metabolism and nutrition disorders | Calcium deficiency***** | 7 | 5.8（2.76-12.19） | 5.8(27.62) | 2.53（0.83） | 5.77(2.74) |
| Nervous system disorders | Apraxia | 7 | 5.69（2.71-11.96） | 5.69(26.89) | 2.5（0.81） | 5.66(2.69) |
| Psychiatric disorders | Emotional poverty | 7 | 5.57（2.65-11.7） | 5.57(26.06) | 2.47（0.8） | 5.54(2.63) |
| Eye disorders | Anisocoria | 7 | 5.35（2.55-11.25） | 5.35(24.63) | 2.41（0.77） | 5.33(2.53) |
| Gastrointestinal disorders | Megacolon***** | 7 | 5.34（2.54-11.22） | 5.33(24.52) | 2.41（0.76） | 5.31(2.53) |
| Psychiatric disorders | Schizoaffective disorder | 7 | 5.05（2.4-10.61） | 5.05(22.61) | 2.33（0.72） | 5.03(2.39) |
| Cardiac disorders | Conduction disorder | 7 | 4.6（2.19-9.66） | 4.6(19.6) | 2.19（0.64） | 4.58(2.18) |
| Nervous system disorders | Aura | 7 | 4.49（2.14-9.43） | 4.49(18.88) | 2.16（0.62） | 4.47(2.13) |
| Nervous system disorders | Marchiafava-Bignami disease | 6 | 302.07（120.64-756.39） | 302.04(1368.18) | 7.84（1.54） | 229.79(91.77) |
| Congenital, familial and genetic disorders | Foetal alcohol syndrome | 6 | 64.49（28.21-147.41） | 64.48(351.29) | 5.92（1.54） | 60.47(26.45) |
| Nervous system disorders | Exaggerated startle response | 6 | 41.29（18.23-93.5） | 41.29(226.1) | 5.31（1.49） | 39.62(17.5) |
| Psychiatric disorders | Rebound psychosis | 6 | 38.01（16.81-85.95） | 38(207.92) | 5.19（1.47） | 36.59(16.18) |
| Skin and subcutaneous tissue disorders | Needle track marks | 6 | 21.5（9.57-48.28） | 21.49(114.66) | 4.4（1.34） | 21.04(9.37) |
| Injury, poisoning and procedural complications | Stab wound | 6 | 21.18（9.43-47.56） | 21.18(112.84) | 4.37（1.34） | 20.74(9.23) |
| Nervous system disorders | Poor sucking reflex | 6 | 16.49（7.36-36.97） | 16.49(85.83) | 4.02（1.25） | 16.23(7.24) |
| Nervous system disorders | Neurodevelopmental disorder | 6 | 15.14（6.76-33.92） | 15.14(78.01) | 3.9（1.22） | 14.92(6.66) |
| Nervous system disorders | Wernicke's encephalopathy | 6 | 13.93（6.22-31.19） | 13.93(70.97) | 3.78（1.18） | 13.74(6.14) |
| Respiratory, thoracic and mediastinal disorders | Apnoeic attack | 6 | 11.93（5.33-26.69） | 11.93(59.35) | 3.56（1.11） | 11.8(5.27) |
| Congenital, familial and genetic disorders | Porphyria acute***** | 6 | 10.89（4.87-24.35） | 10.89(53.28) | 3.43（1.07） | 10.78(4.82) |
| Nervous system disorders | Allodynia | 6 | 10.3（4.61-23.04） | 10.3(49.86) | 3.35（1.04） | 10.2(4.56) |
| General disorders and administration site conditions | Drug tolerance increased | 6 | 8.14（3.64-18.18） | 8.14(37.26) | 3.01（0.91） | 8.08(3.62) |
| Congenital, familial and genetic disorders | Congenital cardiovascular anomaly | 6 | 7.52（3.37-16.8） | 7.52(33.66) | 2.9（0.86） | 7.47(3.35) |
| Gastrointestinal disorders | Intestinal pseudo-obstruction | 6 | 7.35（3.29-16.41） | 7.35(32.65) | 2.87（0.85） | 7.3(3.27) |
| Nervous system disorders | Pleurothotonus | 6 | 7.1（3.18-15.86） | 7.1(31.23) | 2.82（0.82） | 7.06(3.16) |
| Nervous system disorders | Hyporesponsive to stimuli | 6 | 7（3.14-15.63） | 7(30.62) | 2.8（0.81） | 6.95(3.12) |
| Metabolism and nutrition disorders | Poor feeding infant | 6 | 6.66（2.98-14.86） | 6.66(28.65) | 2.73（0.78） | 6.62(2.96) |
| Gastrointestinal disorders | Bezoar | 6 | 6.47（2.9-14.44） | 6.47(27.56) | 2.69（0.76） | 6.43(2.88) |
| Ear and labyrinth disorders | Vestibular disorder | 6 | 5.52（2.48-12.32） | 5.52(22.1) | 2.46（0.65） | 5.5(2.46) |
| Gastrointestinal disorders | Oesophageal haemorrhage***** | 6 | 5.19（2.33-11.59） | 5.19(20.21) | 2.37（0.6） | 5.17(2.32) |
| Investigations | Analgesic drug level increased | 6 | 5.12（2.29-11.41） | 5.11(19.76) | 2.35（0.59） | 5.09(2.28) |
| Hepatobiliary disorders | Fatty liver alcoholic | 5 | 113.87（45.05-287.83） | 113.86(499.85) | 6.67（1.26） | 101.86(40.3) |
| Social circumstances | Pharmaceutical nomadism | 5 | 62.11（25.14-153.48） | 62.11(282.29) | 5.87（1.25） | 58.38(23.63) |
| Social circumstances | Immobilisation prolonged | 5 | 51.99（21.13-127.88） | 51.98(237.11) | 5.63（1.23） | 49.35(20.06) |
| Hepatobiliary disorders | Portal tract inflammation | 5 | 43.48（17.74-106.54） | 43.47(198.47) | 5.38（1.21） | 41.63(16.99) |
| Congenital, familial and genetic disorders | Cryopyrin associated periodic syndrome***** | 5 | 33.92（13.9-82.76） | 33.92(154.26) | 5.04（1.18） | 32.79(13.44) |
| Nervous system disorders | Stiff person syndrome | 5 | 19.44（8.02-47.13） | 19.44(85.72) | 4.25（1.06） | 19.07(7.87) |
| Respiratory, thoracic and mediastinal disorders | Neonatal respiratory depression | 5 | 17.98（7.42-43.56） | 17.98(78.69) | 4.14（1.03） | 17.67(7.29) |
| Psychiatric disorders | Impaired reasoning | 5 | 15.53（6.42-37.57） | 15.53(66.87) | 3.93（0.99） | 15.29(6.32) |
| Injury, poisoning and procedural complications | Chemical poisoning | 5 | 15.14（6.26-36.62） | 15.13(64.97) | 3.9（0.98） | 14.91(6.16) |
| Nervous system disorders | Pyramidal tract syndrome | 5 | 14.19（5.87-34.32） | 14.19(60.41) | 3.81（0.95） | 14(5.79) |
| Injury, poisoning and procedural complications | Carbon monoxide poisoning | 5 | 13.29（5.5-32.11） | 13.28(56.02) | 3.71（0.93） | 13.12(5.43) |
| Social circumstances | Victim of homicide | 5 | 12.49（5.17-30.17） | 12.49(52.15) | 3.63（0.91） | 12.34(5.11) |
| Investigations | Antipsychotic drug level decreased | 5 | 12.08（5-29.18） | 12.08(50.16) | 3.58（0.89） | 11.94(4.94) |
| Nervous system disorders | Seizure cluster | 5 | 11.58（4.79-27.97） | 11.58(47.75) | 3.52（0.87） | 11.45(4.74) |
| Psychiatric disorders | Posturing | 5 | 10.49（4.34-25.32） | 10.49(42.45) | 3.38（0.83） | 10.38(4.3) |
| Surgical and medical procedures | Life support | 5 | 9.14（3.79-22.06） | 9.14(35.92) | 3.18（0.77） | 9.07(3.76) |
| Metabolism and nutrition disorders | Hyperchloraemia***** | 5 | 8.7（3.6-20.98） | 8.69(33.74) | 3.11（0.74） | 8.63(3.58) |
| Psychiatric disorders | Suspiciousness | 5 | 8.66（3.59-20.9） | 8.66(33.59) | 3.1（0.74） | 8.59(3.56) |
| Psychiatric disorders | Borderline personality disorder | 5 | 8.57（3.55-20.67） | 8.57(33.14) | 3.09（0.73） | 8.5(3.53) |
| Psychiatric disorders | Intrusive thoughts | 5 | 8.53（3.53-20.56） | 8.52(32.92) | 3.08（0.73） | 8.46(3.51) |
| Nervous system disorders | Reflexes abnormal | 5 | 8.46（3.51-20.42） | 8.46(32.62) | 3.07（0.73） | 8.4(3.48) |
| Social circumstances | Theft | 5 | 7.81（3.24-18.84） | 7.81(29.47) | 2.96（0.68） | 7.76(3.22) |
| Gastrointestinal disorders | Tongue movement disturbance | 5 | 7.06（2.93-17.03） | 7.06(25.84) | 2.81（0.62） | 7.02(2.91) |
| Injury, poisoning and procedural complications | Electric shock | 5 | 7（2.91-16.88） | 7(25.54) | 2.8（0.62） | 6.96(2.89) |
| Investigations | Electrocardiogram PR prolongation | 5 | 6.95（2.88-16.76） | 6.95(25.29) | 2.79（0.62） | 6.91(2.87) |
| Cardiac disorders | Right ventricular hypertrophy | 5 | 6.19（2.57-14.91） | 6.19(21.6) | 2.62（0.54） | 6.15(2.55) |
| Psychiatric disorders | Social anxiety disorder | 5 | 6.02（2.5-14.49） | 6.02(20.78) | 2.58（0.53） | 5.98(2.48) |
| Nervous system disorders | Brain stem infarction***** | 5 | 5.06（2.1-12.19） | 5.06(16.21) | 2.33（0.41） | 5.04(2.09) |
| Product issues | Product deposit | 5 | 4.92（2.04-11.84） | 4.91(15.51) | 2.29（0.39） | 4.89(2.03) |
| Investigations | Electrocardiogram J wave | 4 | 147.16（51.36-421.67） | 147.15(503.2) | 7（0.89） | 127.66(44.55) |
| Investigations | Osmolar gap abnormal | 4 | 95.65（34.22-267.36） | 95.64(340.57) | 6.44（0.9） | 87.04(31.14) |
| Vascular disorders | Hypotensive crisis | 4 | 58.86（21.44-161.57） | 58.86(214.31) | 5.79（0.88） | 55.5(20.22) |
| Hepatobiliary disorders | Perforation bile duct***** | 4 | 57.11（20.82-156.61） | 57.1(208.06) | 5.75（0.88） | 53.94(19.67) |
| Investigations | Benzodiazepine drug level increased | 4 | 52.41（19.16-143.4） | 52.41(191.23) | 5.64（0.88） | 49.74(18.18) |
| Social circumstances | High risk sexual behaviour | 4 | 41.14（15.12-111.93） | 41.14(150.19) | 5.3（0.86） | 39.48(14.51) |
| General disorders and administration site conditions | Hanging | 4 | 36.44（13.42-98.91） | 36.44(132.8) | 5.13（0.85） | 35.14(12.94) |
| Musculoskeletal and connective tissue disorders | Floppy infant | 4 | 27.33（10.11-73.84） | 27.33(98.64) | 4.73（0.81） | 26.6(9.84) |
| Injury, poisoning and procedural complications | Environmental exposure | 4 | 22.64（8.4-61.02） | 22.64(80.82) | 4.47（0.77） | 22.14(8.21) |
| Pregnancy, puerperium and perinatal conditions | Placental infarction | 4 | 18.66（6.94-50.21） | 18.66(65.58) | 4.2（0.73） | 18.32(6.81) |
| Cardiac disorders | Cardio-respiratory distress | 4 | 17.08（6.35-45.91） | 17.08(59.49) | 4.07（0.71） | 16.8(6.25) |
| Respiratory, thoracic and mediastinal disorders | Cheyne-Stokes respiration | 4 | 16.93（6.3-45.5） | 16.93(58.91) | 4.06（0.71） | 16.65(6.2) |
| Nervous system disorders | Central nervous system necrosis | 4 | 16.71（6.22-44.9） | 16.71(58.05) | 4.04（0.7） | 16.44(6.12) |
| Congenital, familial and genetic disorders | Otospondylomegaepiphyseal dysplasia | 4 | 15.68（5.84-42.12） | 15.68(54.09) | 3.95（0.69） | 15.44(5.75) |
| General disorders and administration site conditions | Injection site thrombosis | 4 | 15.37（5.72-41.27） | 15.36(52.87) | 3.92（0.68） | 15.14(5.64) |
| Social circumstances | Patient uncooperative | 4 | 14.28（5.32-38.32） | 14.28(48.66) | 3.82（0.66） | 14.08(5.25) |
| Congenital, familial and genetic disorders | Microtia | 4 | 12.8（4.77-34.32） | 12.8(42.92) | 3.66（0.62） | 12.64(4.71) |
| Gastrointestinal disorders | Oral discharge | 4 | 12.8（4.77-34.32） | 12.8(42.92) | 3.66（0.62） | 12.64(4.71) |
| Investigations | Body mass index decreased | 4 | 12.11（4.52-32.46） | 12.11(40.25) | 3.58（0.6） | 11.97(4.46) |
| Investigations | Sinus rhythm | 4 | 11.88（4.43-31.85） | 11.88(39.37) | 3.55（0.6） | 11.75(4.38) |
| Nervous system disorders | Brain compression | 4 | 11.35（4.24-30.43） | 11.35(37.32) | 3.49（0.58） | 11.23(4.19) |
| Psychiatric disorders | Neurosis | 4 | 10.51（3.92-28.16） | 10.51(34.05) | 3.38（0.55） | 10.41(3.88) |
| Nervous system disorders | Amnestic disorder | 4 | 10.02（3.74-26.82） | 10.02(32.12) | 3.31（0.53） | 9.92(3.7) |
| Psychiatric disorders | Sleep attacks | 4 | 9.79（3.65-26.2） | 9.78(31.23) | 3.28（0.52） | 9.7(3.62) |
| Psychiatric disorders | Belligerence | 4 | 9.69（3.62-25.94） | 9.69(30.84) | 3.26（0.52） | 9.6(3.58) |
| Gastrointestinal disorders | Faecal vomiting | 4 | 9.54（3.56-25.55） | 9.54(30.28) | 3.24（0.51） | 9.46(3.53) |
| Congenital, familial and genetic disorders | Congenital hydronephrosis | 4 | 6.37（2.38-17.02） | 6.37(17.97) | 2.66（0.32） | 6.33(2.37) |
| Injury, poisoning and procedural complications | Arterial injury | 4 | 6.23（2.33-16.66） | 6.23(17.45) | 2.63（0.31） | 6.2(2.32) |
| Surgical and medical procedures | Finger amputation | 4 | 6.1（2.28-16.31） | 6.1(16.95) | 2.6（0.3） | 6.07(2.27) |
| Nervous system disorders | Circadian rhythm sleep disorder | 4 | 5.87（2.2-15.68） | 5.87(16.05) | 2.55（0.27） | 5.84(2.18) |
| Investigations | Blood fibrinogen increased | 4 | 5.45（2.04-14.56） | 5.45(14.45) | 2.44（0.23） | 5.42(2.03) |
| Psychiatric disorders | Abulia | 4 | 5.42（2.03-14.48） | 5.42(14.33) | 2.43（0.23） | 5.39(2.02) |
| Psychiatric disorders | Scatolia | 3 | 286.95（78.97-1042.71） | 286.93(657.55) | 7.79（0.34） | 220.95(60.81) |
| Social circumstances | Ex-drug abuser | 3 | 119.56（36-397.08） | 119.56(313.51) | 6.73（0.42） | 106.38(32.03) |
| Injury, poisoning and procedural complications | Graft ischaemia | 3 | 110.37（33.4-364.65） | 110.36(291.47) | 6.63（0.42） | 99.05(29.98) |
| Nervous system disorders | Femoral nerve palsy | 3 | 57.39（17.9-184.01） | 57.39(156.81) | 5.76（0.43） | 54.2(16.9) |
| Surgical and medical procedures | Drug withdrawal maintenance therapy | 3 | 54.14（16.92-173.27） | 54.14(148.09) | 5.68（0.42） | 51.29(16.03) |
| Nervous system disorders | Coma acidotic | 3 | 40.99（12.91-130.2） | 40.99(112.24) | 5.3（0.41） | 39.35(12.39) |
| Infections and infestations | Subacute endocarditis***** | 3 | 32.98（10.43-104.27） | 32.98(89.93) | 5（0.39） | 31.91(10.1) |
| Investigations | Pain threshold decreased | 3 | 28.98（9.19-91.42） | 28.98(78.67) | 4.82（0.38） | 28.16(8.93) |
| Congenital, familial and genetic disorders | Choanal atresia | 3 | 24.53（7.8-77.15） | 24.52(66) | 4.58（0.36） | 23.94(7.61) |
| Metabolism and nutrition disorders | Latent autoimmune diabetes in adults | 3 | 23.91（7.6-75.2） | 23.91(64.25) | 4.55（0.36） | 23.35(7.43) |
| Investigations | Analgesic drug level above therapeutic | 3 | 23.71（7.54-74.57） | 23.71(63.69) | 4.53（0.36） | 23.16(7.37) |
| Skin and subcutaneous tissue disorders | Erythrosis | 3 | 22.42（7.14-70.44） | 22.42(59.98) | 4.45（0.35） | 21.93(6.98) |
| Investigations | Urine osmolarity increased | 3 | 21.58（6.87-67.75） | 21.57(57.56) | 4.4（0.34） | 21.12(6.73) |
| Psychiatric disorders | Organic brain syndrome | 3 | 19.13（6.1-59.99） | 19.13(50.53) | 4.23（0.32） | 18.77(5.99) |
| Congenital, familial and genetic disorders | Congenital genitourinary abnormality | 3 | 19（6.06-59.59） | 19(50.17) | 4.22（0.32） | 18.65(5.95) |
| Social circumstances | Physical abuse | 3 | 18.88（6.02-59.19） | 18.88(49.81) | 4.21（0.32） | 18.53(5.91) |
| Surgical and medical procedures | Arm amputation | 3 | 18.75（5.98-58.8） | 18.75(49.45) | 4.2（0.32） | 18.41(5.87) |
| Psychiatric disorders | Excessive masturbation | 3 | 18.28（5.83-57.29） | 18.28(48.07) | 4.17（0.32） | 17.95(5.73) |
| Psychiatric disorders | Dissociative amnesia | 3 | 17.39（5.55-54.48） | 17.39(45.52) | 4.1（0.31） | 17.1(5.46) |
| Congenital, familial and genetic disorders | Coloboma | 3 | 16.78（5.36-52.55） | 16.78(43.75) | 4.05（0.3） | 16.51(5.27) |
| Injury, poisoning and procedural complications | Pneumoconiosis | 3 | 16.3（5.21-51.04） | 16.3(42.37) | 4（0.29） | 16.05(5.13) |
| Investigations | Urine sodium increased | 3 | 15.18（4.85-47.5） | 15.18(39.12) | 3.9（0.28） | 14.96(4.78) |
| Investigations | Blood ethanol increased | 3 | 14.42（4.61-45.09） | 14.42(36.91) | 3.83（0.27） | 14.22(4.55) |
| Injury, poisoning and procedural complications | Wrong drug | 3 | 13.16（4.21-41.13） | 13.16(33.26) | 3.7（0.24） | 13(4.16) |
| Investigations | Myoglobin urine present | 3 | 13.1（4.19-40.94） | 13.1(33.08) | 3.69（0.24） | 12.94(4.14) |
| Surgical and medical procedures | Euthanasia | 3 | 12.75（4.08-39.84） | 12.75(32.07) | 3.66（0.24） | 12.6(4.03) |
| Psychiatric disorders | Echolalia | 3 | 12.64（4.05-39.49） | 12.64(31.74) | 3.64（0.23） | 12.49(4) |
| Psychiatric disorders | Somatic delusion | 3 | 12.42（3.98-38.8） | 12.42(31.1) | 3.62（0.23） | 12.27(3.93) |
| Social circumstances | Crime | 3 | 12.26（3.93-38.3） | 12.26(30.64) | 3.6（0.22） | 12.12(3.88) |
| Eye disorders | Refraction disorder | 3 | 11.91（3.81-37.18） | 11.91(29.6) | 3.56（0.22） | 11.77(3.77) |
| Gastrointestinal disorders | Tongue spasm | 3 | 10.95（3.51-34.18） | 10.95(26.82) | 3.44（0.19） | 10.84(3.47) |
| Neoplasms benign, malignant and unspecified (incl cysts and polyps) | Langerhans' cell histiocytosis***** | 3 | 10.18（3.26-31.74） | 10.17(24.56) | 3.33（0.17） | 10.08(3.23) |
| Investigations | Transferrin saturation decreased | 3 | 9.73（3.12-30.33） | 9.73(23.25) | 3.27（0.16） | 9.64(3.09) |
| Infections and infestations | Botulism | 3 | 9.44（3.03-29.43） | 9.44(22.41) | 3.23（0.15） | 9.36(3) |
| Metabolism and nutrition disorders | Feeding intolerance | 3 | 9.26（2.97-28.86） | 9.26(21.88) | 3.2（0.14） | 9.18(2.94) |
| Injury, poisoning and procedural complications | Complications of transplanted liver | 3 | 9.11（2.92-28.4） | 9.11(21.45) | 3.18（0.13） | 9.03(2.9) |
| Injury, poisoning and procedural complications | Penis injury | 3 | 9.05（2.9-28.22） | 9.05(21.28) | 3.17（0.13） | 8.98(2.88) |
| Investigations | Electrocardiogram QRS complex shortened | 3 | 8.97（2.88-27.95） | 8.97(21.04) | 3.15（0.13） | 8.89(2.85) |
| Respiratory, thoracic and mediastinal disorders | Neonatal aspiration | 3 | 8.94（2.87-27.86） | 8.94(20.96) | 3.15（0.13） | 8.87(2.84) |
| Congenital, familial and genetic disorders | Ductus arteriosus premature closure | 3 | 8.86（2.84-27.61） | 8.86(20.72) | 3.13（0.12） | 8.78(2.82) |
| Psychiatric disorders | Poverty of speech | 3 | 8.46（2.72-26.38） | 8.46(19.57) | 3.07（0.11） | 8.4(2.7) |
| Injury, poisoning and procedural complications | Weaning failure | 3 | 8.34（2.68-25.99） | 8.34(19.22) | 3.05（0.1） | 8.28(2.66) |
| Respiratory, thoracic and mediastinal disorders | Neonatal tachypnoea | 3 | 7.99（2.57-24.9） | 7.99(18.2) | 2.99（0.09） | 7.93(2.55) |
| Nervous system disorders | Facial nerve disorder | 3 | 7.8（2.5-24.29） | 7.8(17.63) | 2.95（0.08） | 7.74(2.49) |
| Congenital, familial and genetic disorders | Hypermobility syndrome | 3 | 7.53（2.42-23.46） | 7.53(16.86) | 2.9（0.06） | 7.48(2.4) |
| Pregnancy, puerperium and perinatal conditions | Threatened labour | 3 | 7.34（2.36-22.85） | 7.34(16.3) | 2.87（0.05） | 7.29(2.34) |
| Investigations | Aspartate aminotransferase decreased | 3 | 7.26（2.33-22.62） | 7.26(16.08) | 2.85（0.05） | 7.22(2.32) |
| Nervous system disorders | Sensorimotor disorder | 3 | 7.02（2.25-21.84） | 7.02(15.36) | 2.8（0.03） | 6.97(2.24) |
| Eye disorders | Vision abnormal neonatal | 3 | 7（2.25-21.79） | 7(15.31) | 2.8（0.03） | 6.95(2.23) |
| Nervous system disorders | Retrograde amnesia | 3 | 6.93（2.23-21.58） | 6.93(15.12) | 2.78（0.03） | 6.89(2.21) |
| Cardiac disorders | Right ventricular dilatation | 3 | 6.82（2.19-21.22） | 6.82(14.78) | 2.76（0.02） | 6.77(2.18) |
| Congenital, familial and genetic disorders | Ear malformation | 3 | 6.7（2.15-20.87） | 6.7(14.46) | 2.74（0.01） | 6.66(2.14) |
| Investigations | Blood bicarbonate increased | 3 | 6.63（2.13-20.63） | 6.63(14.23) | 2.72（0.01） | 6.59(2.12) |
| Investigations | Thyroxine free decreased***** | 3 | 6.52（2.1-20.3） | 6.52(13.93) | 2.7（0） | 6.48(2.08) |
| *****Indicates statistically significant signals in algorithm; ROR, reporting odds ratio; CI, confidence interval; PRR, proportional reporting ratio; χ2, chi-square; IC, information component; IC025, the lower limit of 95% CI of the IC; EBGM, empirical Bayesian geometric mean; EBGM05, the lower limit of 95% CI of EBGM. | | | | | | |
